# Extracellular DNA Release by Undomesticated *Bacillus subtilis* Is Regulated by Early Competence

**DOI:** 10.1371/journal.pone.0048716

**Published:** 2012-11-02

**Authors:** Olga Zafra, María Lamprecht-Grandío, Carolina González de Figueras, José Eduardo González-Pastor

**Affiliations:** Department of Molecular Evolution, Center of Astrobiology (INTA-CSIC), Torrejón de Ardoz, Madrid, Spain; Institut Pasteur, France

## Abstract

Extracellular DNA (eDNA) release is a widespread capacity described in many microorganisms. We identified and characterized lysis-independent eDNA production in an undomesticated strain of *Bacillus subtilis*. DNA fragments are released during a short time in late-exponential phase. The released eDNA corresponds to whole genome DNA, and does not harbour mutations suggesting that is not the result of error prone DNA synthesis. The absence of eDNA was linked to a spread colony morphology, which allowed a visual screening of a transposon library to search for genes involved in its production. Transposon insertions in genes related to quorum sensing and competence (*oppA*, *oppF* and *comXP*) and to DNA metabolism (*mfd* and *topA*) were impaired in eDNA release. Mutants in early competence genes such as *comA* and *srfAA* were also defective in eDNA while in contrast mutations in late competence genes as those for the DNA uptake machinery had no effect. A subpopulation of cells containing more DNA is present in the eDNA producing strains but absent from the eDNA defective strain. Finally, competent *B. subtilis* cells can be transformed by eDNA suggesting it could be used in horizontal gene transfer and providing a rationale for the molecular link between eDNA release and early-competence in *B. subtilis* that we report.

## Introduction

The capacity to release extracellular DNA (eDNA) has been reported in many Bacteria and Archaea [1]–[2], and eDNA is highly abundant in natural environments, such as deep-sea sediments, aquatic environments and biofilms [3]–[5]. The eDNA is released by different mechanisms depending on the microbial species, mostly by lysis and active secretion. In *Pseudomonas aeruginosa* the eDNA is released by lysis likely mediated by prophages or vesicles and is regulated by quorum sensing [6]. In *Staphylococcus epidermidis*, the eDNA is released by lysis of a subpopulation of cells expressing the AtlE autolysin [7]. In *Streptococcus pneumoniae* [8], [9], the induction of natural competence triggers cell lysis and eDNA release. In contrast, in *Neisseria gonorrhoeae* an active type IV secretion system, encoded by the gonococcal genetic island (GGI) is involved in eDNA release [10]. Lastly, release of membrane vesicles that contain DNA has been described in some microorganisms such as *P. aeruginosa* [11] and the hyperthermophilic archea of the order *Thermococcales* [12].

A common biological role has not been proposed for the eDNA, and different functions depending on the microorganism have been reported. eDNA is relevant during the first stages of biofilm formation in *Pseudomonas* [5] [13] [6], *Streptococcus* [14], *Staphylococcus* [7] and *Bacillus cereus* [15]. On the other hand, eDNA can be used as a nutrient in oligotrophic environments [16]. Moreover, eDNA could be involved in gene transfer, diversity increase and evolution [17]–[18] [10]. Using mathematical models, it has been proposed that bacteria that release DNA to the environment could take the place of those who do not because of the benefit of increased genetic diversity [18]. Several groups of Bacteria and Archaea can develop natural competence [19]–[20], and they can be transformed by eDNA [1] [21]. A relationship between competence and eDNA has been observed in *Streptococcus pneumoniae* [8], *N. gonorrhoeae* [22] and *Pseudomonas stutzeri* [23]. *B. subtilis* is naturally competent, and the presence of very low concentrations of eDNA (0.1 µg ml^−1^) has been reported in supernatants from exponential and early stationary phase cultures of the laboratory strain 168 [24]. Interestingly, only the eDNA from late exponential growth supernatants, which was not correlated with cell lysis, could be used in transformation of competent recipient bacteria [24].

At the molecular level, the competence pathway can be divided into early and late stages in *Bacillus subtilis*. In the early stage, ComX, that activates ComP, and CSF that is taken up by oligopeptide permease Opp, act as pheromones (quorum sensing signals). Both signals converge in a common regulator, ComA, that autophosphorylates and accumulates in the cytoplasm. ComA-P activates the operon *srf-comS,* and ComS is the first signal of the late stage. In this late stage, genes for binding and internalization of DNA are transcribed. Other master regulators of the cell activate this late stage of competence, such as DegU, CodY and AbrB that modulate the pathway depending on the physiology and are related to other processes as sporulation and multicellularity [25]. Also, it is known that eDNA production is linked to quorum sensing in *S. pneumoniae* [9] and *P. aeruginosa* [6]. Transformation of cells by eDNA of phylogenetically distant species could activate the defence and repair systems. Given this response to foreign DNA, coordination of the secretion of near homologous DNA in a bacterial community through quorum sensing is likely to be beneficial.

Most of the research in *B. subtilis* has been based in the study of laboratory strains which have been extensively manipulated. As a result, these strains have lost social behaviours that are not essential under laboratory conditions. The studies of the natural or “undomesticated” *B. subtilis* strain 3610 has enabled the discovery of natural behaviours previously unidentified in this bacterium, like the formation of multicellular structures [26] and swarming motility [27]. eDNA release is clearly a social behaviour, mostly induced by quorum sensing signals in a subpopulation of cells. Our work began with an investigation of the ability of the natural strain 3610 to release eDNA during late exponential phase, in a manner independent from cell lysis. We described the connection between eDNA release and competence development. The link of a particular colony phenotype with the defect in eDNA release allowed a genetic screen to search for genes involved in this phenomenon. For the first time, the presence of a peak of eDNA release and its direct connection to early competence development has been shown in this bacterium.

## Materials and Methods

### Bacterial Strains and Media

For this study we used the *Bacillus subtilis* “undomesticated” strain 3610 (natural isolate of *B. subtilis*: NCIB3610; from A. L. Sonenshein and the *Bacillus* Genetic Stock Center (BGSC), Ohio State Univ., Columbus, OH) and PY79 laboratory strain (prototroph, derived from *B. subtilis* strain 168, from P. Youngman, University of Georgia, Athens, GA). Other strains used in this work are described in Table 1.

**Table 1:** Strains used in this work.

Strain	Genotype	Reference
***E. coli***		
DH5α	*fhuA2* Δ*(argF-lacZ)U169 phoA glnV44 Φ80* Δ*(lacZ)M15* *gyrA96 recA1 relA1 endA1 thi-1 hsdR17*	Invitrogen
***B. subtilis***		
NCIB3610	wild type	*Bacillus* Genetic Stock Center
PY79	*swrA srf*	P. Youngman
1A792 (L16648)	168 *lytABC::neo lytD::tet lytE::cat lytF::spc*	from BGSC, n° 1A792 (Margot, 1999)
Bal 376	JH646 Δ*spo0K358::erm*	[68]
Bal 941	JH646 Δ*phrC::tet*	[69]
BD1243	168 *comA::*Tn*917-erm*	[55]
BD1245	168 Δ*comEA::*Tn*917-erm*	[52]
PG677	168 Δ*comK::kan*	[51]
PG679	168 Δ*comGA::erm*	from P.Graumann laboratory
EG168	PY79 *skfABCDEF::tet*	[41]
EG240	3610 *spo0A::erm*	this work
EG245	3610 *srfAA::erm*	[26]
EG385	3610 *amy*E::P*feu*-*lac*Z*-spc*	laboratory collection
EG524	PY79 *sdpABC::erm*	[41]
RL891	PY79 *spo0A::erm*	[26]
GP229	3610 *degU*::mini-Tn*10*	this work
GP230	3610 *comP*:: mini-Tn*10*	this work
GP231	3610 *topA*:: mini-Tn*10*	this work
GP232	3610 *mfd*:: mini-Tn*10*	this work
GP233	3610 *oppA*:: mini-Tn*10*	this work
GP236	3610 Δ*phrC::tet*	this work
GP237	3610 Δ*comK::kan*	this work
GP239	3610 Δ*comGA::erm*	this work
GP240	3610 *comA::*Tn*917-erm*	this work
GP241	3610 Δ*comEA::*Tn*917-erm*	this work
GP304	3610 *nos::erm*	[70]
GP305	3610 spontaneous mutant	this work (SPR-1)
GP306	3610 spontaneous mutant	this work (SPR-2)
GP307	3610 spontaneous mutant	this work (SPR-3)
GP308	3610 Δ*spo0K358::erm*	this work
GP309	3610 *comX-comP*:: mini-Tn*10*	this work
GP310	3610 *oppF*:: mini-Tn*10*	this work
GP311	3610 *yqhG*:: mini-Tn*10*	this work
GP313	3610 *lytABC::neo*	this work
GP314	3610 *lytE::cat*	this work
GP315	3610 *skfABCDEF::tet sdpABC::erm*	this work
GP316	3610 Δ*xkdG::erm*	this work
GP319	3610 spontaneous mutant	this work (SPR-4)

The colony morphology of the strains were analyzed on solid Luria-Bertani (LB) medium (1% Bacto tryptone, 0.5% yeast extract, 1% NaCl) supplemented with 1.5% Bacto agar, incubated at 37°C for 16 h. The LB plates were dried for 16 h before use. For surfactin test, DSM plates were used (8 g/l Bactonutrient broth, 0.1% KCl, 0.012% MgSO_4_, 0.5 mM NaOH, 1 mM Ca(NO_3_)_2_, 0.01 mM MnCl_2_, 1 µM FeSO_4_, 1.5% Bacto agar).

To monitor the DNA release, the strains were grown in minimal medium (MSgg): 5 mM phosphate buffer pH 7, 0.1 M MOPS pH 7, 5 µM FeCl_3_, 2 mM MgCl_2_, 0.7 mM CaCl_2_, 50 µM MnCl_2_, 1 µM ZnCl_2_, 2 µM thiamine, 50 µg ml^−1^ Phe, 50 µg ml^−1^ Trp, 50 µg ml^−1^ Thr, 0.5% glycerol and 0.5% glutamate [28] at 37°C and shaken at 200 rpm. For transformation of *B. subtilis*, cells were grown in Modified Competence Medium (MCM) (100 mM phosphate buffer pH 7, 2% glucose, 3 mM trisodium citrate, 20 µg ml^−1^ ferric ammonium citrate, 0.1% casein hydrolysate,0.2% potassium glutamate) from [29]. Antibiotics concentrations (final) were: 100 µg ml^−1^ spectinomycin, 2 µg ml^−1^ kanamycin, 1 µg ml^−1^ erythromycin plus 25 µg ml^−1^ lincomycin (mls), 50 µg ml^−1^ tetracycline and 7 µg ml^−1^ neomycin.

### *B. subtilis* Natural Transformation

The *B. subtilis* strain 3610 has been previously considered non-competent in laboratory conditions, however, all the competence genes are present and functional in the strain and we have seen in the laboratory that 3610 develops competence with low efficiency under the conditions described here. *B. subtilis* cultures (both 3610 and PY79) grown overnight on liquid LB at 30°C were diluted to A_600nm_ = 0.08 in 10 ml of the Modified Competence Medium (MCM) and were incubated at 37°C and 200 rpm [29]. At the start of stationary phase (A_600nm_ = 1.5–2), 10 µg of genomic DNA were added to 1 ml of the culture. The culture was incubated at least 2 h at 37°C and 200 rpm before plating with the appropriate antibiotic.

### Construction of 3610 Mutants

When mutants were kindly provided in other strains, genomic DNA were isolated and used in transformation of strain 3610 as described. Deletion of *xkdG* gene from *B.subtilis* 3610 genome was achieved by long-flanking homology polymerase chain reaction (LFH-PCR) technique [30]. The deletion/insertion Δ*xkd*G*::mls* was constructed by PCR amplification to obtain approximately 1 kbp from 5′-flanking region of *xkdG* gene with primers P1XkdG (cacttgaggagtggccggagg) and P2XkdG (**attatgtcttttgcgcagtcggc**tcaattgatttcctcctccttgact), while another band of near 1 kbp from 3′-flanking region was amplified using P2XkdG (**cattcaattttgagggttgccag**acgaggtggtcagctcatgctc) and P4XkdG (cccacctcaagcgaaagcccg). The resulting PCR products were then used as primers to amplify the erythromycin-resistance cassette (mls resistance), (hybridizing region in bold) from the plasmid pDG646 [31]. This creates a complete deletion of the *xkdG* gene. The PCR products were used to transform strain PY79 and the mutants were confirmed by PCR. The Δ*xkdG::mls* mutation were then introduced in 3610 strain by transformation and confirmed by PCR analysis.

In the case of strain EG240 construction, strain RL891 was used as donor to transfer the *spo0A::erm* mutation into strain 3610 by the PBS1 phage transduction method [32].

### Measurements of eDNA in Planktonic Cultures

*B. subtilis* strains were grown in MSgg at 30°C overnight and were diluted to A_600nm_ = 0.05 in 50 ml of MSgg at 37°C with agitation at 200 rpm. In each sample, 0.8 ml of the culture were taken and cells were centrifuged (5 min at 5000 rpm in a Eppendorf Centrifuge 5415D), 0.5 ml of the supernatant was used to precipitate the eDNA with sodium acetate and ethanol and resuspended in 20 µl of water [33] The concentration was determined by spectrophotometry (A_260_/A_280_) (S2100 Diode Array Spectrophotometer, WPA Biowave). In the case of *spo0A* and *srfAA* mutant strains, an additional filtration with 0.45 µm Millipore filter was done after centrifugation and before eDNA precipitation.

### Detection of Plasmid pAS32 in the Supernatant

Supernatants from *B. subtilis* 3610 culture at different growth stages were taken and precipitated as described. A PCR, following manufacturer’s instructions, were performed to amplify a specific region of plasmid pAS32 [34] with primers against contig-106 (ABQL01000025): P1-pAS32 (atgtcaccattcataatattacg) and P2-pAS32 (tttaacagcaaagagcttgaag).

### Cloning of eDNA

10 ml of the culture were taken and cells were centrifuged as described (5 min at 5000 rpm in a Eppendorf Centrifuge 5415D). 6 ml of the supernatant were treated with phenol-cloroform-isoamilic alcohol (25∶24:1, pH 8). After that, the sample was precipitated with ethanol and sodium acetate [33]. 3 µg of eDNA were digested with *Sau*IIIA (Roche) following the instructions of the manufacturer. DNA fragments from 400 bp to 3000 bp were selected and purified with the QiaQuick extraction kit (Qiagen). 10 µg of pBluescript SKII+ (Stratagene) were digested with *Bam*HI (Roche) following the manufactureŕs instructions and the 5′ end was dephosphorylated with calf intestinal alkaline phosphatase (CIAP) (Invitrogen). The vector was purified by sodium acetate/ethanol [33] and ligated with eDNA in a 1∶5 ratio vector:insert using DNA ligase T4 (Roche) overnight at 16°C. Ligation was purified by sodium acetate/ethanol and used to transform electrocompetent *E. coli* DH5α cells (Invitrogen). Recombinant clones were sequenced using universal primers M13 (GTAAAACGACGGCCAGTG) and M13-reverse (GGAAACAGCTATGACCAG). Sequence identification was performed using the SubtiList Web Server database (http://genolist.pasteur.fr/SubtiList/) and the BLAST tool [35].

### Test of DNase Activity in 3610 Supernatants

25 µl of supernatant from a *B. subtilis* 3610 culture grown in MSgg were extracted at different time points during growth, added to 2 µg of Molecular Weight Marker III (Roche) and incubated at 37°C for 1 hour. We incubated at the same time a control with 2 µg of Molecular Weight Marker III (Roche) and 25 µl of sterile MSgg. After incubation, 5 µl of each sample was analyzed in an agarose gel to determine the degradation of the sample and, therefore, the presence of DNases.

### Nuclease S1 and DNaseI Assays

To determine if eDNA was double or single strand, it was treated with DNaseI or nuclease S1 respectively. 6 µg of eDNA from *B. subtilis* were incubated with 10 units of recombinant DNaseI-RNase free (Roche), Tris HCl 25 mM pH 8 and 50% glucose at 37°C for 1 h, or with 160 units of nuclease S1 and its buffer (Amersham Pharmacia Biotech) at 37°C for 1 h. In both cases the eDNA was incubated with the same conditions without enzyme as control of the assay and, finally, each sample was analyzed in an agarose gel to determine the degradation of the sample.

### Bacterial Viability, Live/dead Staining and β-galactosidase Assays

We used Live/Dead BacLight dye (Molecular Probes) following the manufactureŕs instructions. A Leica CTR 6000 fluorescence microscope was used. To calculate the percentage of dead bacteria, a minimum of 500 bacteria cells were counted in each sample. Viability was measured by serial dilutions of the culture, that were plated on solid LB. Assays of β-galactosidase activity was performed as described previously [32] [36]. Both intracellular and extracellular β-galactosidase were measured, and the percentage of extracellular β-galactosidase was referred to the total activity (intracellular plus extracellular).

### DNA Microarrays

Microarrays were constructed using 4128 oligonucleotides with 65 bases of length (SIGMA-Genosys), 4106 of which are *B. subtilis* genes and 22 are controls. Oligonucleotides were impressed on Corning® UltraGAPS™ covered with gamma amino propylsilane (GAPS) [37]. Chromosomal and eDNA probes were labelled with fluorescence by direct incorporation of dUTP bond to Cy3 or Cy5 (GE Healthcare) using 2 µg of template and the Klenow fragment (Biolabs) as manufactureŕs recommendations. Products were purified using the QiaQuick purification kit (Qiagen). DNA hybridization were done as previously described by [38]. The microarrays were scanned with Scanner GenePix 4100A (Axon Instrument, Inc.). Images were processed using Genepix Pro 6.0 (Axon Instruments, Inc).

### Transposon Mutagenesis

Transposon mutagenesis was conducted in strain DS1010 [39], a *B. subtilis* 3610 derivative carrying mini-Tn*10* in a temperature sensitive vector. The transposon library was generated as described in Kearns, 2004 [39]. DS1010 was inoculated in LB with spectinomycin and incubated at 22°C for 16 h. The culture was diluted and incubated at 37°C for 4 h. After that, the culture was grown in LB agar plates with spectinomycin. To identify transposon insertions, 4 µg of chromosomal DNA was digested with *Hin*DIII for 4 h at 37°C. The purified reactions were religated in 200 µl and incubated overnight at 15°C. The ligations were purified by microcon tubes, used to transform *E. coli* DH5α and spectinomycin resistance colonies were selected. Each clone was sequenced using primers my050 (GCCGATTCATTAATGCAGG) and my051 (CCCACTTATAAACAAAAGATC) [39]. Sequence identification was performed using SubtiList Web Server database (http://genolist.pasteur.fr/SubtiList/) and the BLAST tool [35].

### Biofilm Formation

Biofilm formation was tested in multiwell plates. The precultures were diluted to A_600nm_ = 0.1 and were grown static in 1 ml MSgg at 30°C during 40 h.

### Flow Cytometry

200 µl of a *B. subtilis* culture were centrifuged and resupended in 1 ml of PBS pH 7.4. Cells were fixed in paraformaldehyde for 2 h. After fixation, cells were washed with PBS, resupended in 20 µl of GTE buffer (50 mM Glucose, 10 mM EDTA at pH 8, 20 mM Tris-HCl at pH 8), and stored at 4°C for further analysis.

For flow cytometric analysis, cells were diluted 1∶100 in PBS and incubated with 0.5 µg µl^−1^ of DAPI (final concentration) for 2 h and measured on a Facs Vantage flow cytometer (Becton Dickinson). For DAPI fluorescence, we used a laser excitation at 360 nm coupled with 424/44 filter. Every sample was analyzed measuring 20,000 events using Cellquest and the study of the overstrike of DAPI intensity among the wild type and the mutant samples were realized using CXP analysis. Cell aggregates were discriminated on the basis of height versus area of the fluorescence intensity of the cells. The experiment was repeated twice.

## Results

### eDNA Production in Bacillus Subtilis 3610

**Figure 1:**
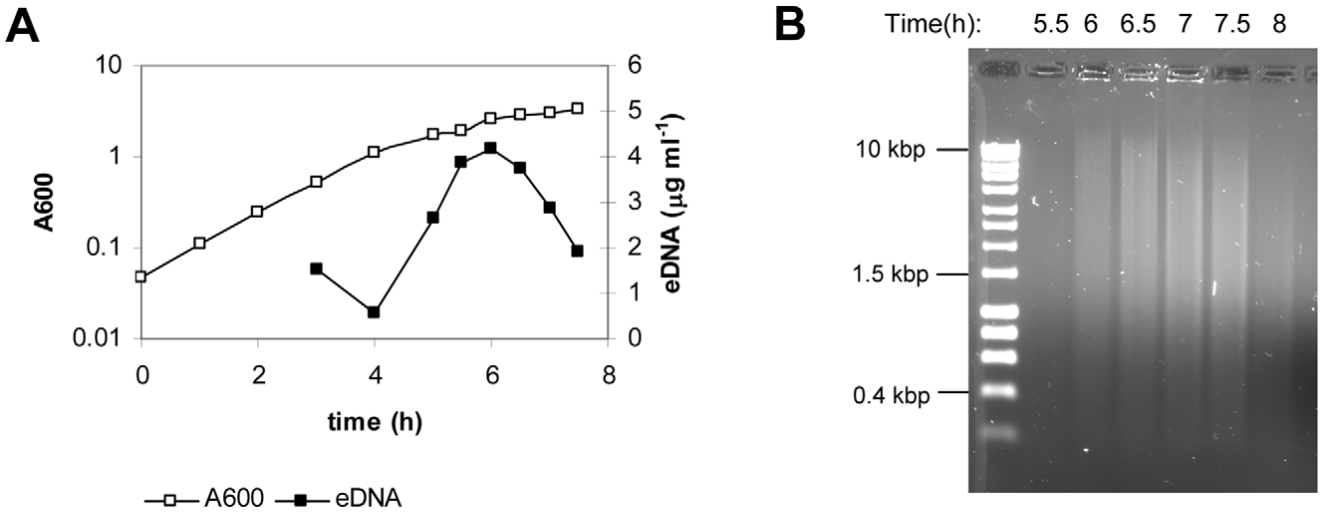
eDNA production in *B. subtilis* 3610. A. Batch culture of strain 3610 in MSgg at 37°C with aeration. A600 refers to the absorbance of the culture at 600 nm, and eDNA refers to the eDNA concentration in the culture supernatant. Data presented are representative of results obtained in, at least, three independent experiments. B. eDNA from another culture of strain 3610 was electrophorized on an agarose gel, the same volume of precipitated eDNA was loaded in each well.

We wished to follow up on the previously reported presence of low concentration eDNA (0.1 µg/ml) in culture supernatants of the *B. subtilis* laboratory strain 168 [24], in an undomesticated *B. subtilis* strain, 3610, that exhibits social behaviours lost in laboratory strains. This strain was grown in liquid minimal medium with aeration and eDNA was precipitated at different time points from the supernatants and quantified by spectrophotometric measurement. The *B. subtilis* 3610 cultures contained a low basal level of eDNA at the beginning of exponential-phase but a large amount of eDNA was released at late exponential-phase, which was immediately followed by a fast decrease in the eDNA concentration (Fig. 1). The graph in Fig. 1A corresponds to one representative experiment, the maximal concentration of eDNA in the supernatant varied between 2.4–6 µg ml^−1^, however the peak of DNA accumulation was always observed at the end of exponential-phase, when cells already arrested growth, thus eDNA accumulation was not the result of a higher density of cells. The abrupt decrease of eDNA levels was not related to the presence of DNases in the supernatant (Fig. S1) but we cannot rule out DNases associated to cell surface. eDNA was run in an agarose gel (Fig. 1B) and it was found to be fragmented in a size range from 10 Kbp to 400 bp. eDNA production was also assessed in the laboratory strain of *B. subtilis* PY79, in contrast, its presence did not correspond with a defined pattern and the timing of the peak of maximal eDNA accumulation varied among experiments (Fig. S2).

**Figure 2:**
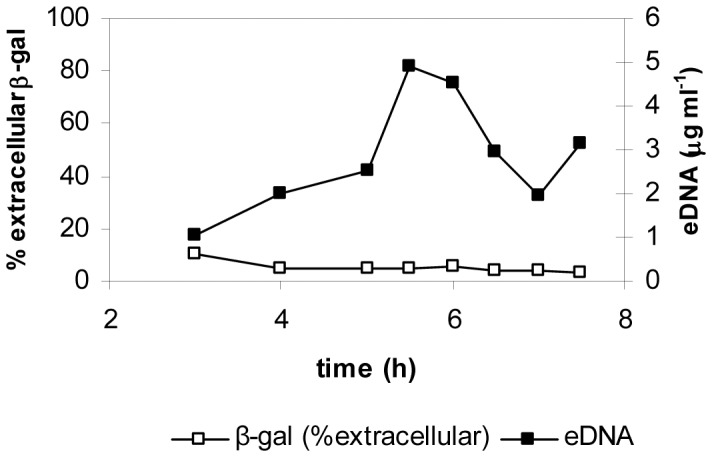
eDNA production in *B. subtilis* 3610 is not caused by lysis. The percentage of extracellular β-galactosidase was measured in cultures of 3610 *amyE*::P*feuA*-*lacZ* (EG385). The strain was grown in liquid MSgg at 37°C with aeration. β-gal refers to the percentage of β-galactosidase activity outside the cell and eDNA refers to the eDNA concentration in the culture supernatant. Data presented are representative of results obtained in, at least, three independent experiments.

The release of eDNA at a precise time point during the growth cycle of strain 3610 suggested that a specific mechanism could be involved. The measurement of β-galactosidase activity in the supernatant of *lacZ*-containing strains has been used to deduce if the release of eDNA occurs by lysis in other bacteria [6] [8]. To shed light on the mechanism involved in the production of eDNA in *B. subtilis*, we used the strain 3610 *amyE*::P*feu*-*lacZ* (EG385) which showed a constitutive expression of β-galactosidase (unpublished data), to check if lysis occurred concomitantly with the eDNA release. We measured the β-galactosidase activity in both supernatant and inside cells in growing cultures of this strain. The fraction of extracellular β-galactosidase activity during growth remained constant even during the peak of eDNA accumulation and values were too low to link eDNA release to cell lysis (Fig. 2). To exclude that the supernatant could have an inhibitory effect on β-galactosidase activity we measured it in eDNA producing cells, in the presence and in the absence of the supernatant, and no significant difference was observed (data not shown). Moreover, other experiments excluded lysis as the mechanism involved in eDNA release. First, we measured viable cells in late exponential phase cultures by plating serial dilutions on LB agar, and viability remained constant at the point of maximum eDNA accumulation. In addition, the same samples used for viability were stained with the Live/Dead BacLight kit (Molecular Probes) and the percentage of dead cells was quantified in a fluorescence microscope, which was always below 5% even at the peak of eDNA release (Fig. S3A). On the other hand, we tested if genes involved in lysis of *B. subtilis* cells could also be related to eDNA release. Therefore, we introduced into the strain 3610 mutations in autolysins *lytABC* and *lytE* [40] (Fig. S3B and S3C) and in the cannibalism clusters *skf* and *sdp* (Fig. S3D), which encode antibiotic peptides involved in the killing of non-sporulating cells in sporulating cultures of *B. subtilis* [41]–[42]. The resulting mutant strains released eDNA similarly to the wild type strain, although in the case of cannibalism mutant the maximum is shifted, probably because of a different growth rate. Thus the lysis related to these autolysins and the cannibalism phenomenon was not connected to eDNA release. Furthermore, Shingaki *et al* found some evidence of the presence of two bands, 13 and 50 kbp, of chromosomal DNA in the early exponential phase of *B. subtilis* 168, linked to the expression of genes encoding capsid proteins from the PBSX prophage (*xkdG*, *M* and *K*) [43]. Therefore, we constructed a mutation in *xkdG*, one of the capsid-encoding genes in the strain 3610 and tested the eDNA presence. We found that the Δ*xkd*G mutant produced eDNA as the wild type (Fig. S3E), thus the phenomenon described by Shingaki *et al* [43] in *B. subtilis* 168 is not related to the eDNA secretion in 3610. In summary, we concluded that, in *B. subtilis*, the natural isolate 3610 releases eDNA during a short time in late-exponential phase, which is not related to cell lysis, therefore, a specific machinery could transport the eDNA outside the cells.

### eDNA Characterization

**Figure 3:**
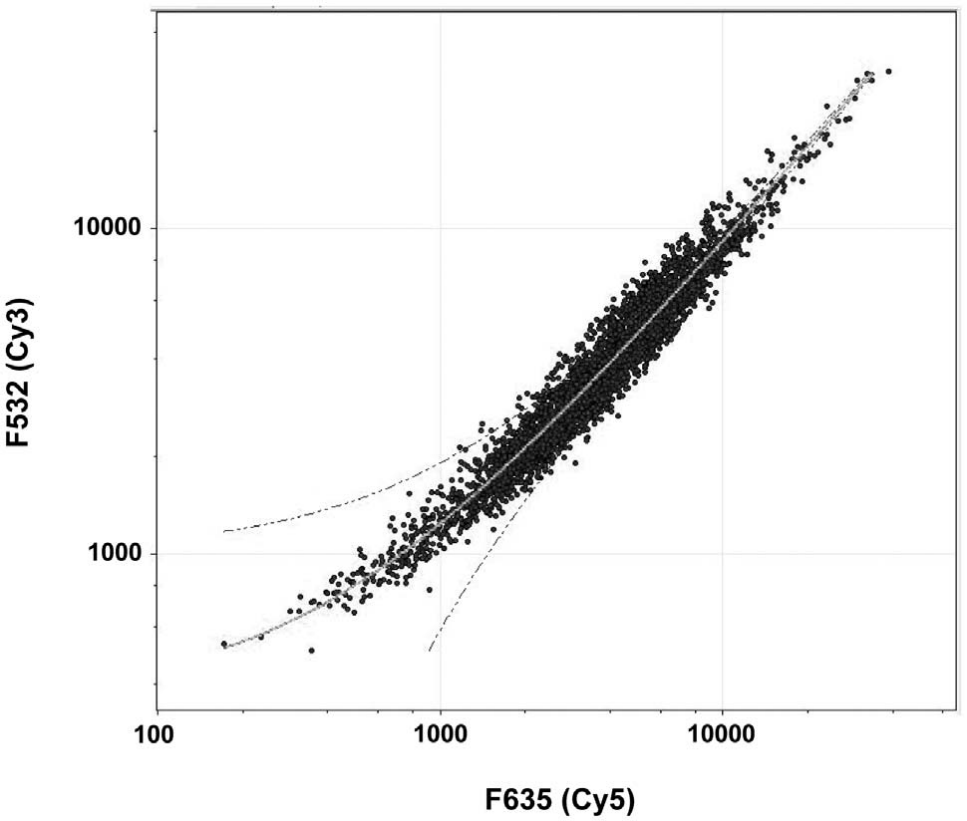
eDNA corresponds with the complete genome of *B. subtilis*. eDNA was labelled with Cy3 (F532) and genomic DNA with Cy5 (F635) and hybridized on an oligonucleotide-based microarray of *B. subtilis* 168.

In other microorganisms extracellular DNA corresponds to chromosomal DNA [7] [44]. In order to characterize the gene composition of the eDNA released by *B. subtilis* 3610 we used an oligonucleotide microarray. eDNA and chromosomal DNA from the strain 3610 were labelled with Cy3 dye (F532) and Cy5 dye (F635) respectively, as described in Materials and Methods. The labelled samples were hybridized on an oligonucleotide microarray based on the *B. subtilis* 168 strain sequence [37]. As it is shown in Fig. 3, all spots hybridized equally, thus we concluded that eDNA corresponds with the complete genome of *B. subtilis* and that there are no regions differentially represented. Additionally, the strain 3610 contains the plasmid pAS32 [34], and its presence in the supernatant at the maximum point of eDNA release was tested by PCR. We conclude that pAS32 was also secreted (data not shown).

Next, we investigated whether the eDNA could contain mutations, as a source of genetic variability. The eDNA was partially digested with the *Sau*IIIA restriction enzyme and cloned in the pBluescript plasmid. We sequenced the inserts from 22 clones, and the sequences of 19 of them were identical to the database sequences of the *B. subtilis* 168 strain. The sequence of the inserts of the 3 remaining clones had slight differences than could be explained because of the sequence differences between strains 3610 and 168 [34] (unpublished data). Therefore we deduced that error-prone polymerases are not involved in eDNA production and that the eDNA corresponds with genomic DNA at the sequence level.

We wondered if the eDNA was single or double stranded. Given that it was possible to digest and to clone the eDNA, we assumed that most of the eDNA could be double stranded. Digestion of the eDNA with DNaseI (RNase free) or with nuclease S1, which degrades only single-strand DNA, confirmed that view, because eDNA was completely digested only by DNaseI but not with nuclease S1 (Fig. S4). We discarded that RNA were the predominant nucleic acid secreted as we used a DNase-RNase free.

### Screening for Genes Involved in eDNA Production

**Figure 4:**
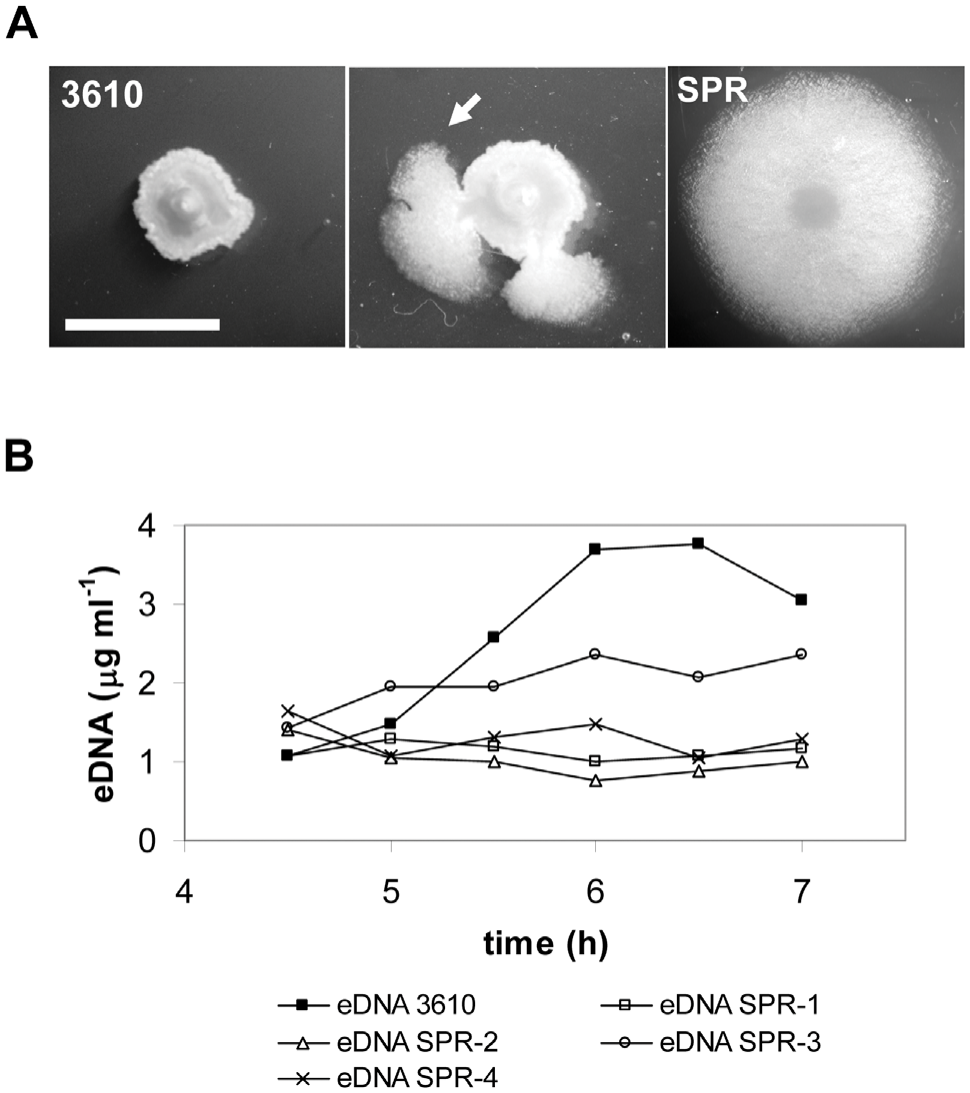
Spread colony phenotype of 3610 spontaneous mutants. A. Differences in colony morphology between 3610 (left) and SPR, a spontaneous spread mutant (GP305, right) growing on LB. The picture in the middle corresponds to a 3610 colony with two branches of spread mutants (arrow). Bar represents 1 cm. B. eDNA production of wild type (3610) and several isogenic spread mutants: SPR-1 (GP305), SPR-2 (GP306), SPR-3 (GP307) and SPR-4 (GP319). Strains were grown in MSgg at 37°C with aeration (growth was similar for all the strains). eDNA refers to the eDNA concentration in the culture supernatant. Data presented are representative of results obtained in two independent experiments.

A common problem for studying the genetics of the eDNA production in different microorganisms is to develop a screening strategy. Colonies formed by strain 3610 on solid rich media have a compact morphology and they do not spread on the surface (Fig. 4A). We noticed that around 5% of the colonies growing on LB solid medium developed branches which were able to spread more on the surface than the original colony. In addition, cells isolated from the branches inherited this phenotype, and we called them “spread mutants” (SPR) (Fig. 4A). We wondered if differences in the physical appearance of the colony between wild type and spontaneous spreading mutants correlated with the ability to produce eDNA. Interestingly, as it is shown in Fig. 4B, four independent spread mutants were affected in eDNA production. All of them lacked the specific eDNA peak released at late exponential phase.

Based on the relation between the spread morphology and the absence of eDNA, we set up a visual screening method to search for genes involved in eDNA production. A transposon mutagenesis library was generated in a *B. subtilis* 3610 derivative carrying a mini-Tn*10*, only 3% of clones lacked the mini-Tn*10* insertion. The library was grown on LB agar plates and 19,000 colonies were screened for spread colonies. 34 colonies were initially selected and their morphology was revised before checking their ability to produce eDNA. Most of them (88%) showed defects in eDNA production as compared with wild type morphology colonies (unpublished data). To identify the insertion sites of the transposons, a protocol was carried out as described in Materials and Methods, and mutations from selected strains were used to retransform on *B. subtilis* 3610 background to confirm that the defect in eDNA production was linked to the transposon insertion and not due to the emergence of additional mutations in the chromosome. Finally, we selected 23 mutant strains which had spread morphology and defects in eDNA production, listed in Table 2 (eDNA production graphs of the transposon mutants used in this work are shown in Fig. S5). It is worth noting that twelve of the transposon insertions were located in the same sequence position into *oppA*, one into *oppF*, four in the *comXP* region (different sites) and one in *degU*, all of them are genes related to competence and quorum sensing functions. Also, we found two transposon insertions at different positions of *mfd* and one in *topA*, both genes related to DNA metabolism. Two additional transposon insertions were located at the same site in *yqhG*, a gene with unknown function.

**Table 2:** Genes involved in eDNA release identified by the transposon library mutagenesis.

Number of clones	Gene	Insertion	Function
12	*oppA*	CGCTTGACC	Oligopeptide permease, involved in quorum sensing, sporulation and competence
1	*oppF*	GCTCACATA	ATP binding protein of the Opp transporter (quorum sensing, sporulation, competence)
1	Deletion *comX-comP*	From AAAGCTTAT (*com*X) to TGCTGAGCA (*com*P)	ComX, pheromone involved in competence. ComP, histidine kinase sensor (two component system), phosphorilation of ComA, competence
3	*comP*	AACTTTGAC GGTGTAACT GGTCAAGCA	ComP, histidine kinase sensor
1	*degU*	GTTGTGGTC	Competence regulator (two component system). Essential for expression of ComK (regulator required for the transcription of genes involved in DNA uptake and integration. Also involved in motility
2	*mfd*	GACCCTTAC TGCAGGCTA	DNA repair (displaces RNA polymerase stalled at a lesion). DNA homologous recombination
1	*topA*	TGTGAAGTG	DNA topoisomerase I
2	*yqhG*	GGCCCCGCG	unknown

In summary, most of the transposon insertions producing spread morphology in the 3610 colonies are also defective in eDNA production, and they interrupt genes related to competence, quorum sensing and DNA metabolism.

### Early Competence Genes are Involved in eDNA Production

**Figure 5:**
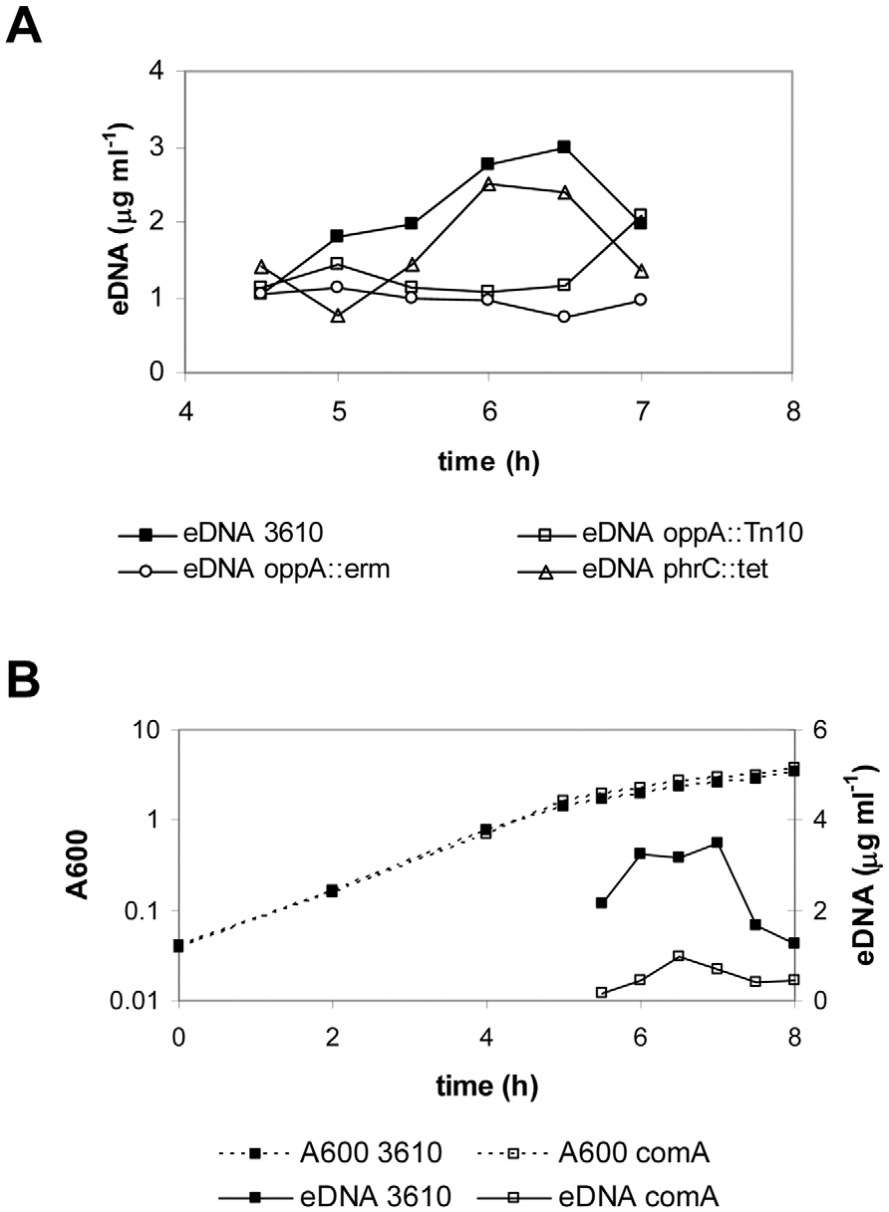
Effect of *oppA*, *phrC* and *comA* in eDNA production. A. The graph shows the eDNA concentration on the culture supernatants of wild type 3610 and the mutants *oppA::*mini*-*Tn*10* (GP233), a insertional mutant in *opp*A; Δ*oppA*::*erm* (GP308), a deletion mutant in *opp*A; and the deletion mutant Δ*phrC* (GP236); (growth was similar for all the strains). B. Growth and eDNA levels of *com*A mutant (GP240) compared with wild type 3610.

Mutants affected in eDNA production point to the link of this phenomenon to competence. Transposon insertions inactivate genes directly affecting early events in competence like *oppA*, *oppF*, *comP*, and *comX*. The *oppA* and *oppF* genes belong to the *opp* operon, which encodes an oligopeptide permease required for the import of the quorum sensing pentapeptide CSF, encoded by the *phrC* gene. CSF contributes to the activation of the ComA transcription factor [19], a response regulator required for competence development [19] [45]. The *oppA* gene was repeatedly targeted at the same position in independent transposition events in contrast to the other genes. To test if the absence of eDNA in the *oppA*::mini-Tn*10* strain (GP233) was due to the effect of this specific insertion of the transposon or to a general defect in *oppA*, we checked the effect on eDNA production of an *oppA* deletion mutant strain (GP308), carrying the mutation Δ*spo0K358::erm*, a deletion from codon 18 to codon 127 of the *oppA* gene. The phenotype observed was the same in both strains (Fig. 5A), thus we conclude that a defect in *oppA* gene affects eDNA production. We also tested a *phrC* mutant, unable to produce CSF, to understand if the eDNA production was related to the role of the oligopeptide permease in the import of this signal. The eDNA production in a Δ*phrC* mutant (GP236) was slightly affected but was still high compared with the wild type strain, depending on the experiment it ranged from 50 to 80% of the production in 3610 (Fig. 5A). Thus, we deduce that the role of the oligopeptide permease (Opp) in eDNA production is partially related to the CSF-regulated pathway and we cannot exclude that other signalling pathways could also be involved in eDNA production.

**Figure 6:**
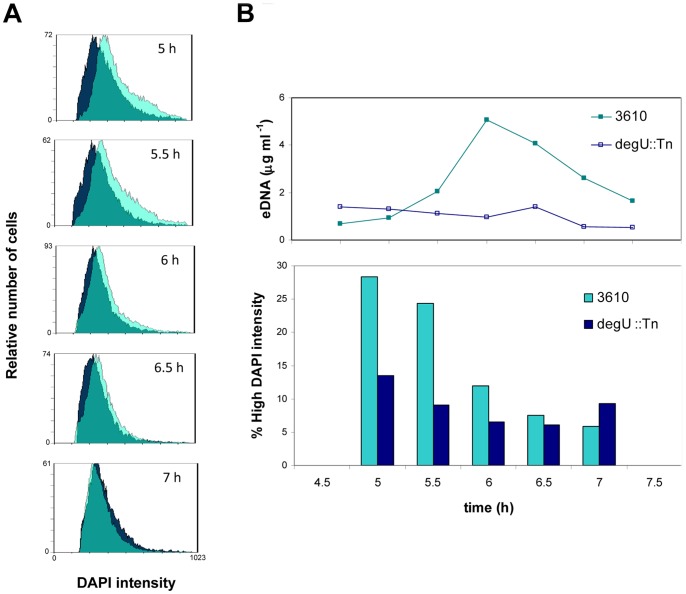
Flow cytometry analysis. A. Graphs show the overlapping of the DAPI intensity profiles of the wild type 3610 (light green) and the *degU*::mini-Tn*10* mutant (GP229) (dark blue), at several time points during growth. B. Upper side graph represents eDNA levels of 3610 and *degU*::mini-Tn*10* mutant. In the lower graph, bars represent percentage of cells with DAPI intensity higher than the mean in *B. subtilis* 3610 and *degU*::mini-Tn*10* mutant (GP229). Only one representative experiment is shown of two independent experiments. Growth was similar in both cases.

In addition to the Opp permease, ComP and ComX also have a regulatory role in early competence. The ComX pheromone stimulates activity of the histidine kinase ComP [46], which activates ComA by phosporylation [47]. Thus, we also checked for eDNA production in a *comA* mutant (GP240) (Fig. 6B), and a *srfAA* mutant (EG245) (Fig. S6A) which also affects *comS*, the main target of ComA [48]–[49]. Both mutants were impaired in eDNA production, underlining the relation of this phenomenon to early competence. Additionally, to analyse if surfactin has a direct role in eDNA production and colony spread morphology we checked its presence in some mutants in DSM plates. Neither *comA* mutant nor *srfAA* released surfactin, but strains *degU*, *oppA* and *phrC* mutants and all spread mutants (SPR-1, 2, 3 and 4) produced it (data not shown). Therefore, surfactin is not directly related in eDNA production.

Next, we wondered if late competence was involved in eDNA production. Thus, we tested different mutants affected in late competence, such as *comK* (GP237), the main regulator of late competence development [50]–[51] and some late competence genes encoding components of the DNA transport machinery such as *comEA* (GP241) [52] and *comGA* (GP239) [53]. All the tested late competence genes released eDNA like the control strain (Fig. S6B and Fig. S6C), excluding their role in this phenotype. In summary, our results prove that eDNA production is under the control of genes involved in early but not in late competence development. Moreover, we found that the master regulator of sporulation, Spo0A, which also controls competence [54]–[55], was defective in eDNA production (Fig. S6D).

### A Subpopulation of Cells with High DNA Content could Release the eDNA

eDNA production implies that a higher level of DNA replication occurs in all or a group of cells in the population. In fact, a gene involved in DNA replication, *topA*, was identified in the screen for mutants impaired in eDNA production. Using fluorescence microscopy and DAPI staining, we observed less DNA staining in the eDNA production defective strains *degU*::mini-Tn*10* (GP229) and *oppA*::mini-Tn*10* (GP233). In order to quantify this observation and to investigate if it was affecting to all or a subpopulation of cells, flow cytometry techniques were used. A wild type and an eDNA defective strain (*degU*::mini-Tn*10*, GP229) were grown in MSgg medium and samples were taken at different time points to be fixed and stained with DAPI (Materials and Methods). The eDNA was also quantified from the supernatant. A total of 20,000 cells from each sample were analyzed for DAPI intensity and the results between wild type and mutant strain were compared. The distribution of cell size was similar for both populations and the distribution of DAPI intensity inside the population was not linked to a particular cell size as shown in the density plots (FSC-H vs DAPI intensity) (Fig. S7). The DAPI basal intensity was calculated from a stationary-phase population (7 hours) of a *degU*::mini-Tn*10* (GP229) mutant. “High DAPI intensity” was referred to higher intensities than this reference. We observed a great difference in the percentage of cells with high DAPI intensity between wild type and mutant strains between 5 h and 6 h of growth, which corresponds with the phase of eDNA production, at the end of exponential growth, thus cells do not duplicate actively, and both strains have the same growth rate (Fig. 6). Also it should be noted that, in the late growth time points (Fig. 6, 6.5 h and 7 h) both the mutant and the wild type show similar DAPI staining. These results suggest that only a subpopulation of cells in the eDNA producing strain highly replicates its DNA before the peak of eDNA production. This group of cells, with high DAPI intensity, was reduced in the eDNA defective strain.

### eDNA can Contribute to Horizontal Gene Transfer in *B. subtilis* Populations

We next sought to determine a functional role for eDNA in *B. subtilis* 3610. eDNA has been reported to contribute to the cohesion of cells during biofilm formation in *Pseudomonas aeruginosa* [5]–[6] and in *Bacillus cereus* [15]. Thus, we first tested the ability of eDNA defective strains to form biofilms. Four strains, the wild type (3610), the spread mutant (GP305), the *oppA*::mini-Tn*10* mutant (GP233) and the laboratory strain (PY79, which produces a weak biofilm) were grown in minimal media (MSgg) for 40 h at 30°C without shaking. A significant difference between the biofilm formed by the strains 3610 and PY79 was observed, as expected, but the spread mutant and *oppA*::mini-Tn*10* mutant (eDNA deficient) formed the same robust biopellicules as the strain 3610 (Fig. S8). In addition, in MSgg solid medium the eDNA defective mutants formed wrinkled colonies with aerial structures identical to those formed by the undomesticated strain 3610 [26] (Fig. S8). Clearly, eDNA production in *B. subtilis* is not required for biofilm formation.

**Figure 7:**
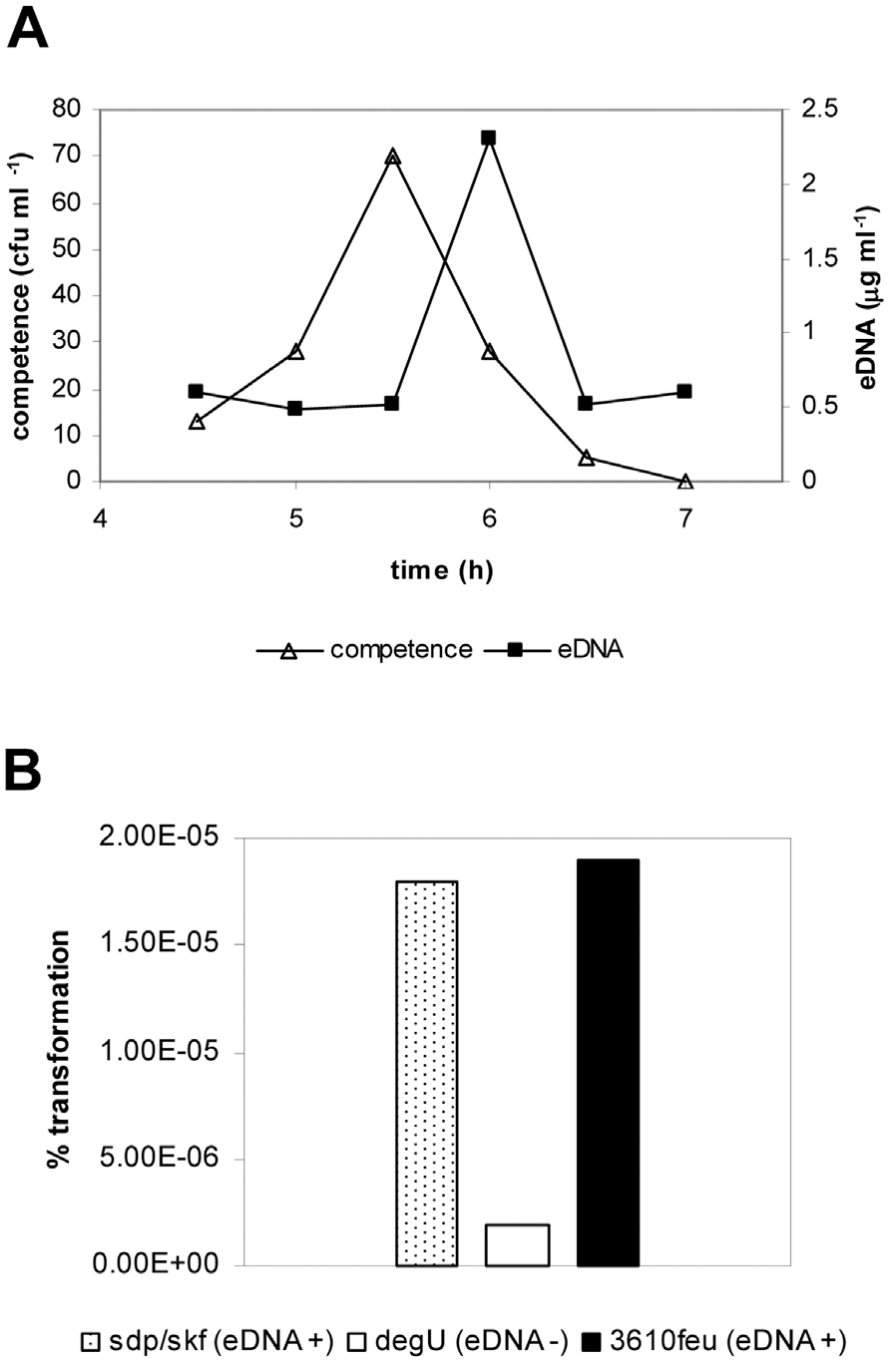
Role of the eDNA in horizontal gene transfer. A. Competence development and eDNA production during *B. subtilis* 3610 growth in MSgg medium, 37°C. B. Transformation of eDNA from culture supernatants in late exponential phase in *B. subtilis* PY79. “skf/sdp” stands for the cannibalism mutant (GP315), degU is the *degU*::mini-Tn*10* mutant strain (GP229) and “3610feu” stands for *amy*E::P*feu*-*lac*Z strain (EG385). One representative experiment of two is shown.

The connection of eDNA production to competence raised the question if this DNA could be used to share genetic information in the population. The eDNA defective mutants obtained in this work were affected in competence, which was expected for competence mutants, but a competence defect was also observed in the spontaneously-arising spread mutant (Fig. S9). In addition, we observed that the timing of eDNA production and competence acquisition overlapped during the growth cycle with competence becoming evident at most 30 minutes prior to eDNA production (Fig. 7A). We then checked if *B. subtilis* cells could be transformed by eDNA. Culture supernatant collected at the maximum peak of eDNA production from different strains, was filtered to avoid spore and cell contamination, and added to competent *B. subtilis* cells (strain PY79). The supernatants came from strains containing antibiotic markers to be selected after transformation: 3610 *amyE::*P*feu-lacZ* (EG385) a wild type strain with antibiotic marker, 3610 *degU::*mini-Tn*10* strain (GP229) that is eDNA deficient, and 3610 *skfABCDEF::tet sdpABC::erm* (GP315), a strain defective in cannibalism behaviour, as a control to detect if the eDNA generated by cannibalism-mediated lysis could be transferred in the population. We transformed the same volume of the filtered supernatant of the different strains, but the eDNA quantity varies (300 ng for *amyE*::P*feu-lac*Z and *skfABCDEF::tet sdpABC::erm*, and 180 ng for *degU*). The transformation frequency of the eDNA from these supernatants was measured, and only strains with eDNA production yielded transformants, and the frequency was similar in the wild type and in the cannibalism mutant (Fig. 7B). Therefore, *B. subtilis* competent cells could be transformed by eDNA not released by lysis in early stationary phase, and it could be used to share genetic information in the population.

## Discussion

In this work, the natural isolate NCBI 3610 was selected to explore eDNA release, since this undomesticated strain has been reported to be a better model to study natural social behaviours [26]. We have characterized at the molecular level the eDNA production and its regulation, which is linked to the early competence pathway. Strain 3610 releases a significant and specific double stranded eDNA production associated with late exponential-phase. A significant peak of double stranded eDNA was released by the strain 3610 during late exponential phase. In contrast, this peak of eDNA release was not observed in the laboratory strains 168 and PY79. A very low amount of eDNA is produced by strain 168 during exponential and stationary phase [24], and we showed that the eDNA released by strain PY79 is very variable and non specific of any growth phase. The concentration of eDNA in the supernatant of a *B. subtilis* 3610 culture is variable as described in other microorganisms [6], but it is noteworthy that it is always associated with late exponential-phase. Our work confirms that the fast decrease of eDNA production after the maximum peak could not be explained by supernatant DNase activity, thus the presence of cell surface DNases or eDNA interaction with cells could be plausible reasons for the eDNA decrease in the medium. We have characterized the eDNA from *B. subtilis* by molecular techniques such as hybridization with oligonucleotide microarrays and sequencing, and it was confirmed that the eDNA corresponds to the complete chromosome and is not mutated, which indicates that it is synthesized by a normal replicative machinery, not an error-prone polymerase.

Two main mechanisms of eDNA production have been described: i) lysis, for instance in *Pseudomonas aeruginosa* [6], and ii) specific release, as in *Neisseria* [10]. In the natural strain 3610, our data clearly excludes eDNA production as a result of a lytic process: i) a *lac*Z marker was introduced in the *B. subtilis* chromosome and the percentage of extracellular versus intracellular β-galactosidase activity, as indicator of lysis, was measured. We did not observe significant cell lysis during the growth cycle in *B. subtilis* not even specifically at the time of the peak of eDNA production in contrast to the results presented by Allesen-Holm for *Pseudomonas aeruginosa* [6]. ii) In addition, the numbers of viable cells were measured, and the ratio of live/dead cells was estimated by staining of samples during the growth cycle of *B. subtilis* and cell lysis was also discarded, and iii) we constructed mutants affected in genes related with bacterial lysis as autolysins (*lytABC* and *lytE*) and cannibalism clusters (*sdp* and *skf*), and the eDNA was still released as in wild type strain. On the other hand, Shingaki *et al* [43] found several evidences that prophage genes (*xkdG*, *M* and *K*) are involved in the release of 13 and 50 kbp DNA corresponding to the chromosome of *B. subtilis* 168. Thus, we tested the eDNA production on a *xkdG* mutant but it was similar to the wild type, indicating that the eDNA release observed in our experiments is not induced by the products of those prophage genes. Therefore, by different experimental approaches we have shown that the eDNA release in *B. subtilis* strain 3610 is not caused by lysis but rather by a specific release mechanism as it has been described for other Gram positive bacteria as *Neisseria* [10]. We propose a specific, active mechanism involved in eDNA production in *B. subtilis*, but additional research will be required to determine if a type IV secretion system or a translocase mediates the release.

The findings that the eDNA is not the result of cell lysis and that it corresponds to the whole *B. subtilis* genome implies a high level of replication of the chromosome, in addition to what is required for cellular division, to explain the high amount of eDNA released. In fact, by DAPI staining of the chromosome and flow cytometry analysis we observed a higher proportion of cells from strain 3610 containing more DNA as compared with an eDNA production mutant. Thus, in a culture of the strain 3610 a subpopulation of cells likely has a higher replication rate than in the defective mutant. Also in other bacteria, as *S. pneumoniae* and *P. aeruginosa,* it is described that the eDNA is also released from a subfraction of the cell population [7] [6].

The strain 3610 allowed us to develop a visual screening method to look for genes involved in eDNA production in a transposon mutant library. Spontaneous mutants of this strain have a spread morphology growing on solid rich medium and are defective in eDNA release. We searched for spread colonies in a transposon mutant library of the strain 3610, based on the assumption that eDNA production could be linked to this morphology, which was true for most of the selected mutants. We do not know how the colony morphology relates to defective eDNA release but future characterization of the molecular pathways of eDNA production and the consequences of taking up eDNA may shed light on this. Transposon mutants affected in three classes of genes were identified in this screening. i) Genes related to competence and quorum sensing (*oppA, oppF, comXP, degU*). Interestingly, in around 50% of the mutants selected the transposons were inserted at the *oppA* gene, thus the oligopeptide permease (Opp) could be an essential step in the phenomenon. Opp is an ABC transporter involved in the active import of signal oligopeptides into the cell [56]–[57]. We discard Opp as DNA transporter because it is involved in active oligopeptide import to the cell and DNA transport requires a very different pore size, thus we support a more likely function related with the pathway of competence or quorum sensing. ii) Genes involved in DNA metabolism as the gene *mfd* that encodes a transcription-repair coupling factor [58] and *topA* that encodes a DNA topoisomerase. These genes could be related to the higher replication rate of strains producing eDNA shown by flow cytometry analysis. It is possible that we did not find more replicative enzymes because of deleterious effects of these mutants. In the case of *topA*, a relationship with competence is reported, since it is regulated by ComK [59]. And iii) a gene encoding an unknown protein, *yqhG*, that should be further investigated. This screening has a limitation, since it is based on the link of the spread morphology and the defect in eDNA released. However other genes involved in this phenomenon could have no effect on colony morphology. For instance, we did not identify mutations clearly affecting genes related with an active transport of DNA. This could be explained by the bias of our screening or by a lethal effect of a mutation affecting the release of the eDNA, which could be accumulated in the cell in the absence of the active transporter.

The results from the transposon mutagenesis reveal a clear link between the regulation of early competence and eDNA production. OppA is the sensor subunit of the oligopeptide permease of *B. subtilis* involved in competence and quorum sensing [46]. The CSF (competence and sporulating factor) is a pentapeptide that links OppA to activate competence pathway through *com*A [19]. Thus, we tested if the role of OppA in eDNA production was related with CSF function. We observed that a CSF mutant, Δ*phrC*, was partially affected in eDNA production. Bongiorni *et al* [60] described that PhrF works synergistically with CSF in *B. subtilis*, and other peptides are involved in the regulation of early stage competence genes, like PhrK required for the expression of *srfA* [61]. Therefore, the Opp oligopeptide permease is involved in eDNA production partially throughout CSF and, probably, other quorum sensing factors still unknown. Opp and ComXP are implicated in eDNA production and both converge in the *comA* gene, which encodes the main regulator of early competence, and in fact, we observed the absence of eDNA in a *comA* mutant. To further characterize the molecular relationship between competence and eDNA production we also tested genes involved in late stages of competence such as *comK* and other related to DNA uptake machinery (*comEA* and *comGA*). Those genes are not affecting eDNA production, therefore at this stage there is a divergence in competence and eDNA production pathways and the DNA uptake machinery can be discarded as related in eDNA extrusion. In summary, regulators of the early stage of competence are controlling eDNA release, which connects the eDNA phenomenon to the competence pathway in *B. subtilis* for the first time. An interesting point is the possible implication in eDNA release of the *comS* gene, whose expression could be affected by a polar effect of the *srfAA* mutation (*comS* gene is inside the *srfAB* gene in a different open reading frame, [62]). We can exclude a direct role of surfactin in both colony morphology and eDNA release, because surfactin production was not affected in other eDNA defective mutants such as *oppA*, *degU, phrC* and also the spread mutants, only *srfAA* and *comA* mutants were affected in surfactin production. The main mechanism described for ComS is the control of ComK levels. ComS prevents the binding of the proteolytic complex MecA-ClpCP with ComK which is not degraded [63]. Another role for ComS cannot be excluded, for instance, it has been shown that phosphorylated DegU is also degraded by MecA-ClpCP protease [64]. Therefore, we suggest that ComS could also prevent degradation of DegU-P, which is in agreement with the defect in eDNA release observed in a *degU* mutant.

What is the role of the eDNA in *Bacillus subtilis*? In many microorganisms the eDNA has been shown to be involved in biofilm formation [5]–[6] [14] [7]. In *B. subtilis* this role can be discarded, since strains defective in eDNA production were forming biofilms as a wild type strain. Our results strongly support that the eDNA could be used for gene transfer in *B. subtilis* populations. First, we have shown a clear regulatory link between competence and eDNA production, as previously discussed. In addition, we observed the absence of competence in the spread mutant strain, defective in eDNA production. Transposon-mutant strains are also affected in competence, some in early stages of the competence pathway, and others such as *topA*, which is defective in competence and its expression is enhanced by ComK [59]. Second, we provide physiological evidence of a coincidence in time of both phenomena in the case of the natural *B. subtilis* strain 3610. And third, the eDNA from *B. subtilis* is functional in horizontal gene transfer (HGT), since the eDNA from supernatants of wild type cultures can be used in *B. subtilis* transformation. In agreement, the eDNA released by strain 168 during late exponential phase, at the time of a peak of competence, can be also useful for transformation [24]. Therefore, our results strongly support a role for the eDNA in HGT as it is proposed in other competent microorganisms as *Neisseria* and *Streptococcus* [22] [8], and provide a rationale for the molecular link between eDNA release and early competence. However, an additional role of the eDNA as a template for DNA repair can not be excluded [65]–[66], which could be a part of a wider function as template for biodiversity through HGT.

In a population of *B. subtilis*, cells starting sporulation initially produce activated Spo0A, the master regulator of sporulation, at a low level that is sufficient to activate genes involved in specialized processes indirectly related to the sporulation phenomenon, such as cannibalism and biofilm formation. However, genes involved specifically in spore formation are only expressed when Spo0A accumulates to very high levels [67]. Similarly, in the early stage of competence it has been described that the ComX-ComP-ComA quorum sensing system is affecting genes involved in cell membrane, modification of the extracellular environment, and also cannibalism and biofilm formation. All these processes are not directly required for the binding, uptake, and processing of transforming DNA, which are activated in a later stage, but they are needed to prepare the cell population for the development of competence [45]. We have shown that eDNA production is also controlled by the ComX-ComP-ComA system, and competent cells can be transformed by eDNA. Thus, the production of eDNA is part of the competence development which could be originated for transforming DNA secreted from cells of the same population or closely related strains.

In conclusion, we have demonstrated the specific production of eDNA in the late exponential-phase of *B. subtilis* 3610 growth, which corresponds to the whole genome. Molecular evidence of the link between regulation of both eDNA release and competence is provided, and further investigation will be required to search for the eDNA secretion mechanism.

## Supporting Information

Figure S1 **DNase assay in 3610 cultures.** Supernatant culture was taken at different times of growth and DNase activity was measured as described in Experimental Procedures. A. Treatment of a DNA marker with different culture supernatants, CSP stands for “culture supernatant”. B. Batch culture of strain 3610 in MSgg at 37°C and eDNA concentration in the supernatant.
(TIF)

Figure S2 **eDNA production in** ***B. subtilis*** **PY79.** Batch culture of PY79 in MSgg at 37°C with aeration. A600 refers to the absorbance of the culture at 600 nm, and eDNA refers to the eDNA concentration in the culture supernatant; in this case, one representative experiment from at least three, is shown to note the unsteady production of eDNA in strain PY79.
(TIF)

Figure S3 **eDNA in** ***B. subtilis*** **3610 is not released by lysis.** A. Death percentage of cells (upper graph) and viable cells (lower graph) in a culture of the strain 3610, quantified by Live/Dead staining and fluorescence microscopy. B. Batch cultures of autolysin mutants, *lytABC::neo* (GP313) and *lytE::cat* (GP314) and eDNA concentration in the culture supernatant. C. Batch culture of a cannibalism mutant (GP315) and eDNA concentration in the culture supernatant. D. Batch culture of a mutant in a capsid protein from prophage PBSX, Δ*xkdG::erm* (GP316), and eDNA concentration in the culture supernatant. Mutants were isogenic with strain 3610. Strains were grown in MSgg at 37°C with aeration. A600 refers to the absorbance of the culture at 600 nm, and eDNA refers to the extracellular DNA concentration in the culture supernatant. Data presented are representative of results obtained in, at least, two experiments.
(TIF)

Figure S4 **eDNA of** ***B. subtilis*** **3610 is mostly double-stranded.** eDNA was isolated from 3610 cultures and digested with DNaseI or nuclease S1. As it could be seen, only DNaseI, but not nuclease S1 degrades all the eDNA, deducing that it is mostly double stranded.
(TIF)

Figure S5 **Effect of transposon mutations in eDNA production.** A. Growth and eDNA levels of *yqhG* (GP311) and *degU* (GP229) transposon mutants compared with 3610. B. Growth and eDNA levels of *comXP* mutant (GP309) compared with wild type 3610. C. Growth and eDNA levels of *oppA* (GP233), *topA* (GP231) and *oppF* (GP310) transposon mutants compared with wild type. D. Growth and eDNA levels of *comP* mutant (GP230) compared with strain 3610. E. Growth and eDNA levels of *mfd* mutant (GP232) compared with wild type strain.
(TIF)

Figure S6 **Effect of** ***srfAA,*** **late competence genes and** ***spo0A*** **in eDNA production.** A. Growth and eDNA levels of *srfAA* mutant (EG245) compared with 3610. B. Growth and eDNA levels of *comK* (GP237) and *comGA* (GP239) mutants compared with wild type 3610. C. Growth and eDNA levels of *comEA* mutant (GP241) compared with wild type. D. Growth and eDNA levels of *spo0A* mutant (EG240) compared with strain 3610.
(TIF)

Figure S7 **Flow cytometry analysis: density plots.** It is shown the distribution of DAPI intensity versus cell size (FSC-H) into the population used in the analysis, from both wild type strain and *degU*::Tn mutant (GP229). Colours represent number of cells. A. 5 hours time point. B. 6.5 hours time point.
(TIF)

Figure S8 **Effect of eDNA on biofilm formation.** *B. subtilis* 3610, PY79, SPR-1 mutant (GP305) and *oppA::*mini*-*Tn*10* (GP233) were grown in MSgg liquid medium at 30°C without shaking during 40 h, and in MSgg solid medium at 37°C 16 h. The presence of a biofilm is visualized as an opaque pellicle on the top of the liquid medium. Negative control refers to media without inoculation.
(TIF)

Figure S9 **Competence assays in eDNA production mutants.** 10 mg of genomic DNA with an antibiotic marker were transformed in several strains of *B. subtilis* and the colonies forming units were quantified to measure competence.
(TIF)
