# Supplementary figures and images for: Extracellular DNA Release by Undomesticated Bacillus subtilis Is Regulated by Early Competence
Source: PLoS One. 2012 Nov 2;7(11):e48716. doi: 10.1371/journal.pone.0048716 (PMC3487849; doi:10.1371/journal.pone.0048716)

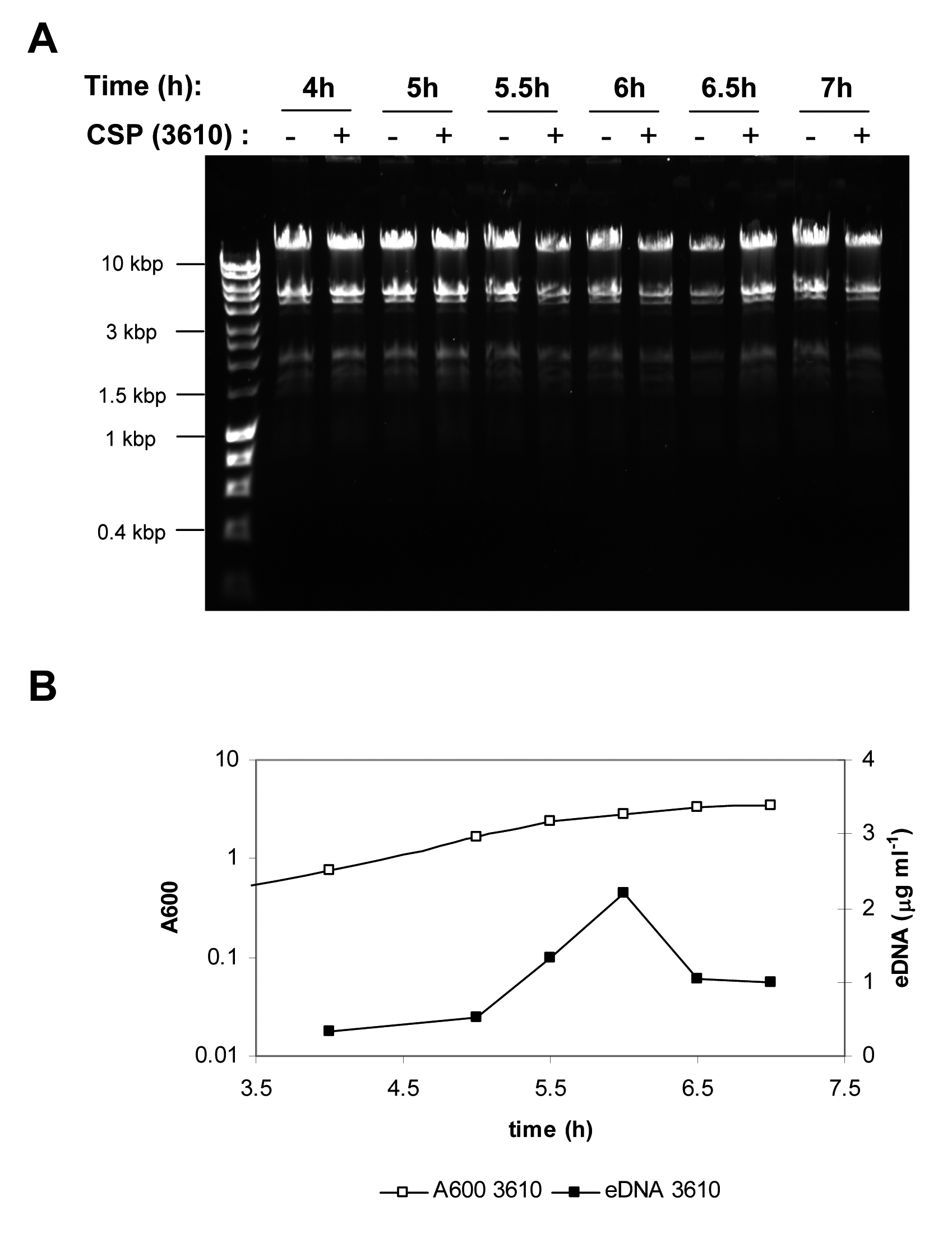

Supplement: Figure S1 — DNase assay in 3610 cultures. Supernatant culture was taken at different times of growth and DNase activity was measured as described in Experimental Procedures. A. Treatment of a DNA marker with different culture supernatants, CSP stands for “culture supernatant”. B. Batch culture of strain 3610 in MSgg at 37°C and eDNA concentration in the supernatant. (TIF) [file pone.0048716.s001.tif]

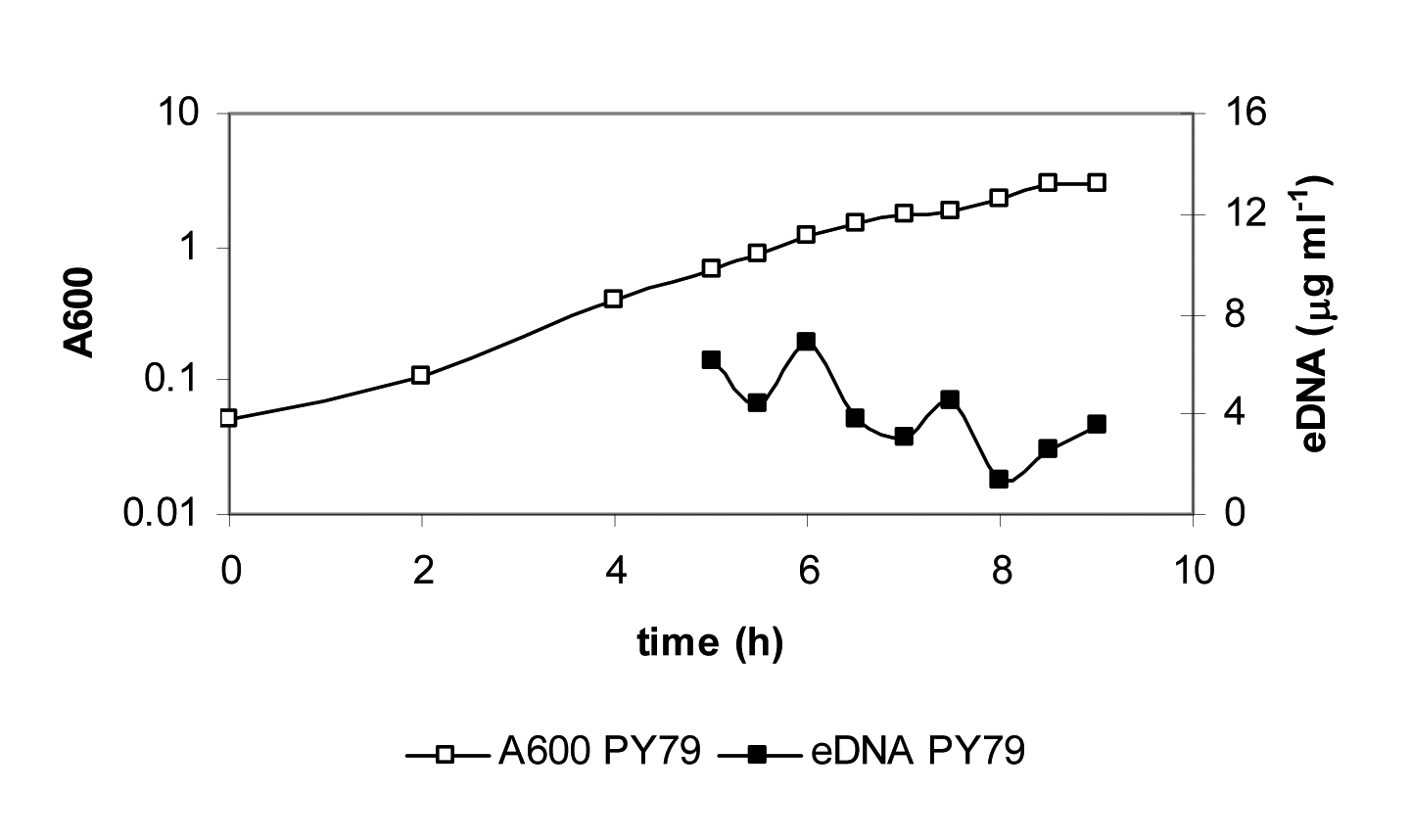

Supplement: Figure S2 — eDNA production in B. subtilis PY79. Batch culture of PY79 in MSgg at 37°C with aeration. A600 refers to the absorbance of the culture at 600 nm, and eDNA refers to the eDNA concentration in the culture supernatant; in this case, one representative experiment from at least three, is shown to note the unsteady production of eDNA in strain PY79. (TIF) [file pone.0048716.s002.tif]

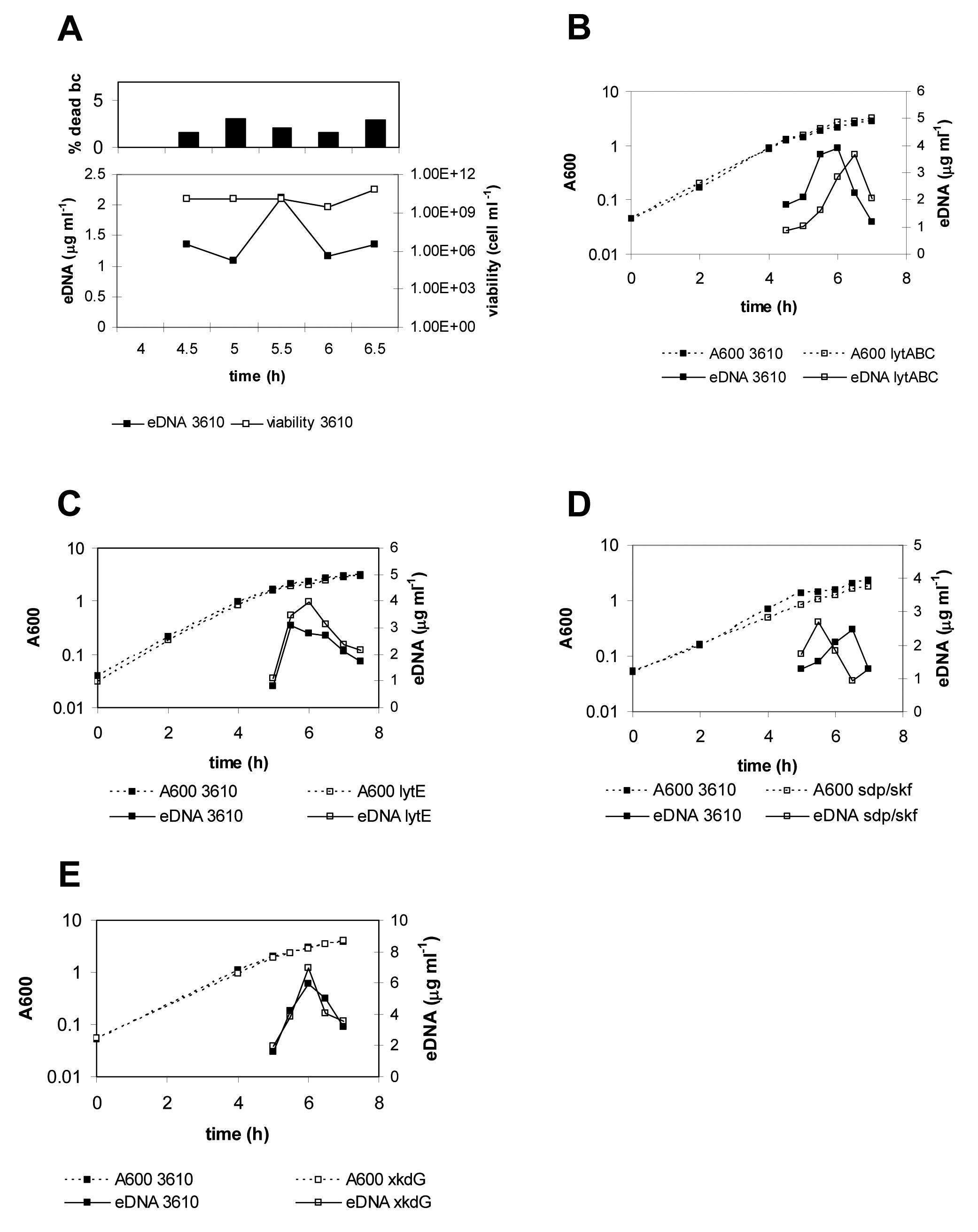

Supplement: Figure S3 — eDNA in B. subtilis 3610 is not released by lysis. A. Death percentage of cells (upper graph) and viable cells (lower graph) in a culture of the strain 3610, quantified by Live/Dead staining and fluorescence microscopy. B. Batch cultures of autolysin mutants, lytABC::neo (GP313) and lytE::cat (GP314) and eDNA concentration in the culture supernatant. C. Batch culture of a cannibalism mutant (GP315) and eDNA concentration in the culture supernatant. D. Batch culture of a mutant in a capsid protein from prophage PBSX, ΔxkdG::erm (GP316), and eDNA concentration in the culture supernatant. Mutants were isogenic with strain 3610. Strains were grown in MSgg at 37°C with aeration. A600 refers to the absorbance of the culture at 600 nm, and eDNA refers to the extracellular DNA concentration in the culture supernatant. Data presented are representative of results obtained in, at least, two experiments. (TIF) [file pone.0048716.s003.tif]

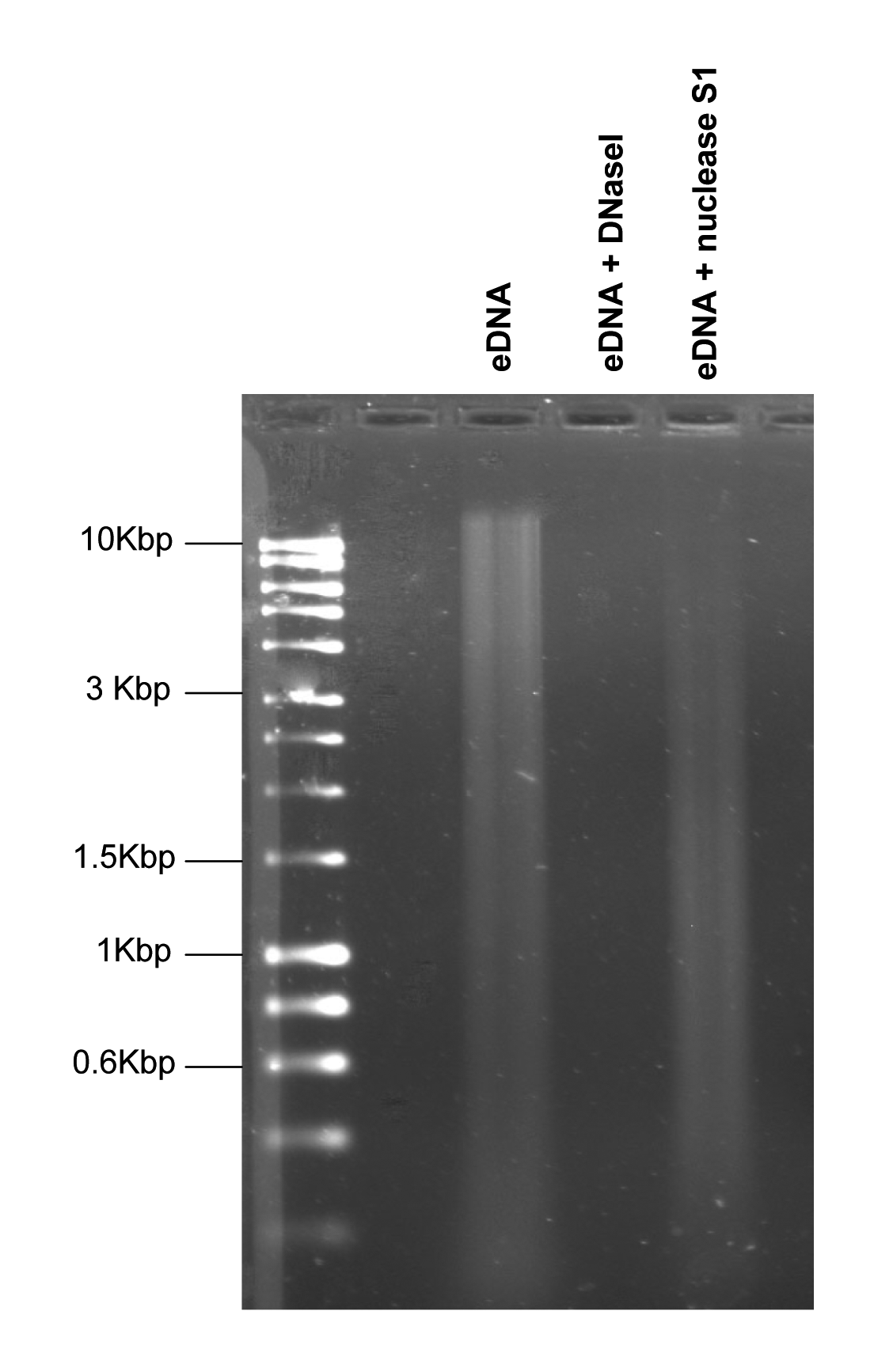

Supplement: Figure S4 — eDNA of B. subtilis 3610 is mostly double-stranded. eDNA was isolated from 3610 cultures and digested with DNaseI or nuclease S1. As it could be seen, only DNaseI, but not nuclease S1 degrades all the eDNA, deducing that it is mostly double stranded. (TIF) [file pone.0048716.s004.tif]

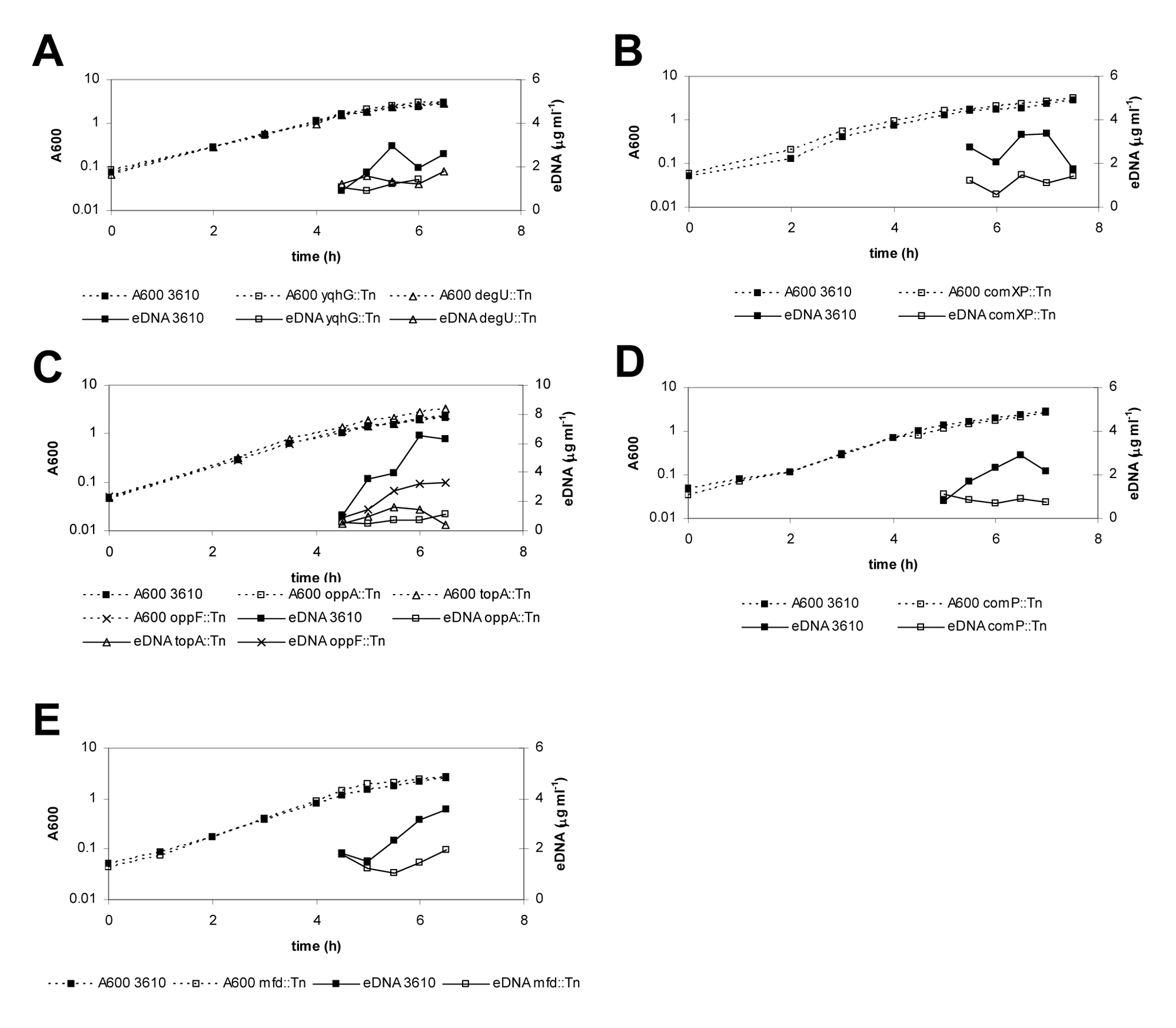

Supplement: Figure S5 — Effect of transposon mutations in eDNA production. A. Growth and eDNA levels of yqhG (GP311) and degU (GP229) transposon mutants compared with 3610. B. Growth and eDNA levels of comXP mutant (GP309) compared with wild type 3610. C. Growth and eDNA levels of oppA (GP233), topA (GP231) and oppF (GP310) transposon mutants compared with wild type. D. Growth and eDNA levels of comP mutant (GP230) compared with strain 3610. E. Growth and eDNA levels of mfd mutant (GP232) compared with wild type strain. (TIF) [file pone.0048716.s005.tif]

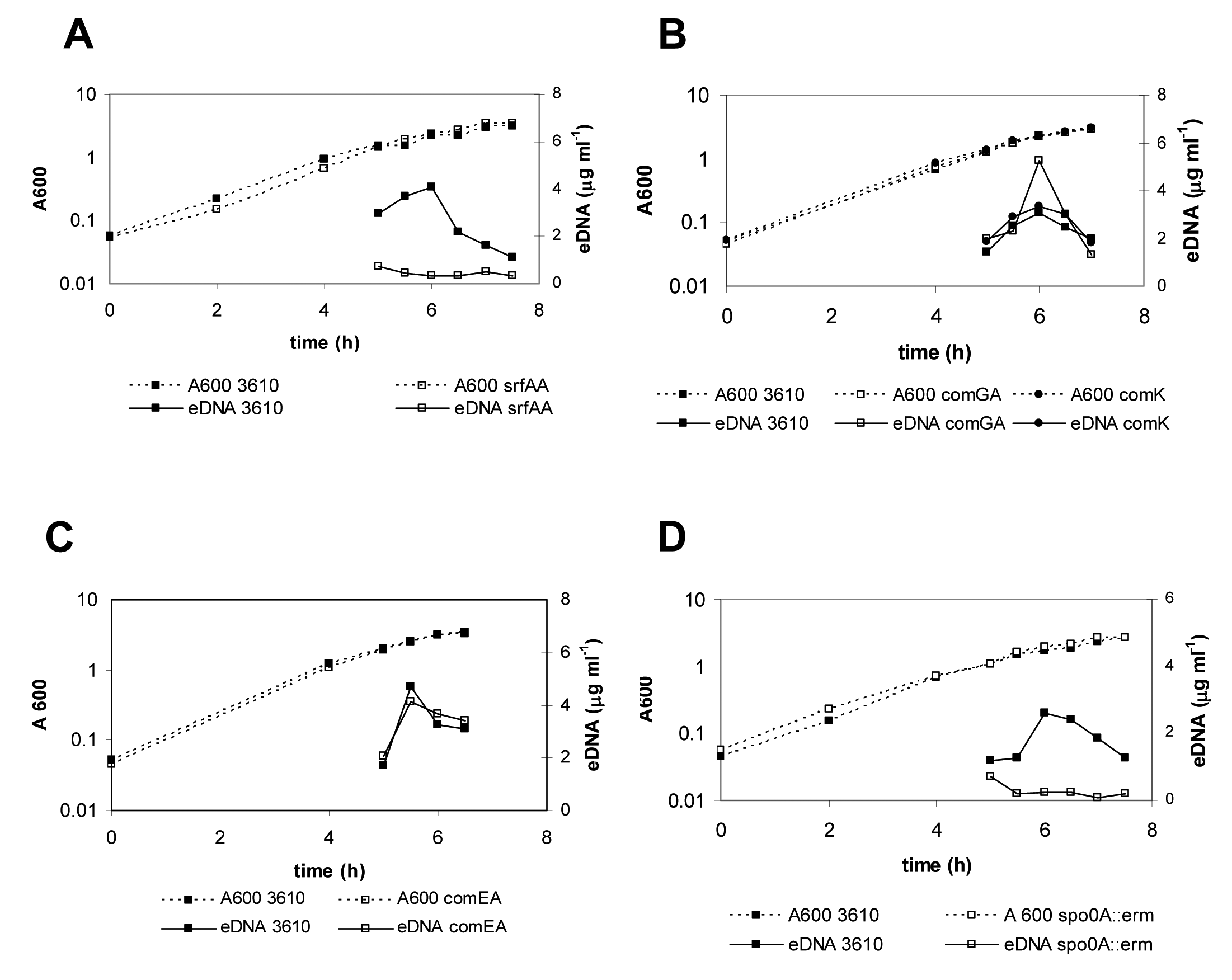

Supplement: Figure S6 — Effect of srfAA, late competence genes and spo0A in eDNA production. A. Growth and eDNA levels of srfAA mutant (EG245) compared with 3610. B. Growth and eDNA levels of comK (GP237) and comGA (GP239) mutants compared with wild type 3610. C. Growth and eDNA levels of comEA mutant (GP241) compared with wild type. D. Growth and eDNA levels of spo0A mutant (EG240) compared with strain 3610. (TIF) [file pone.0048716.s006.tif]

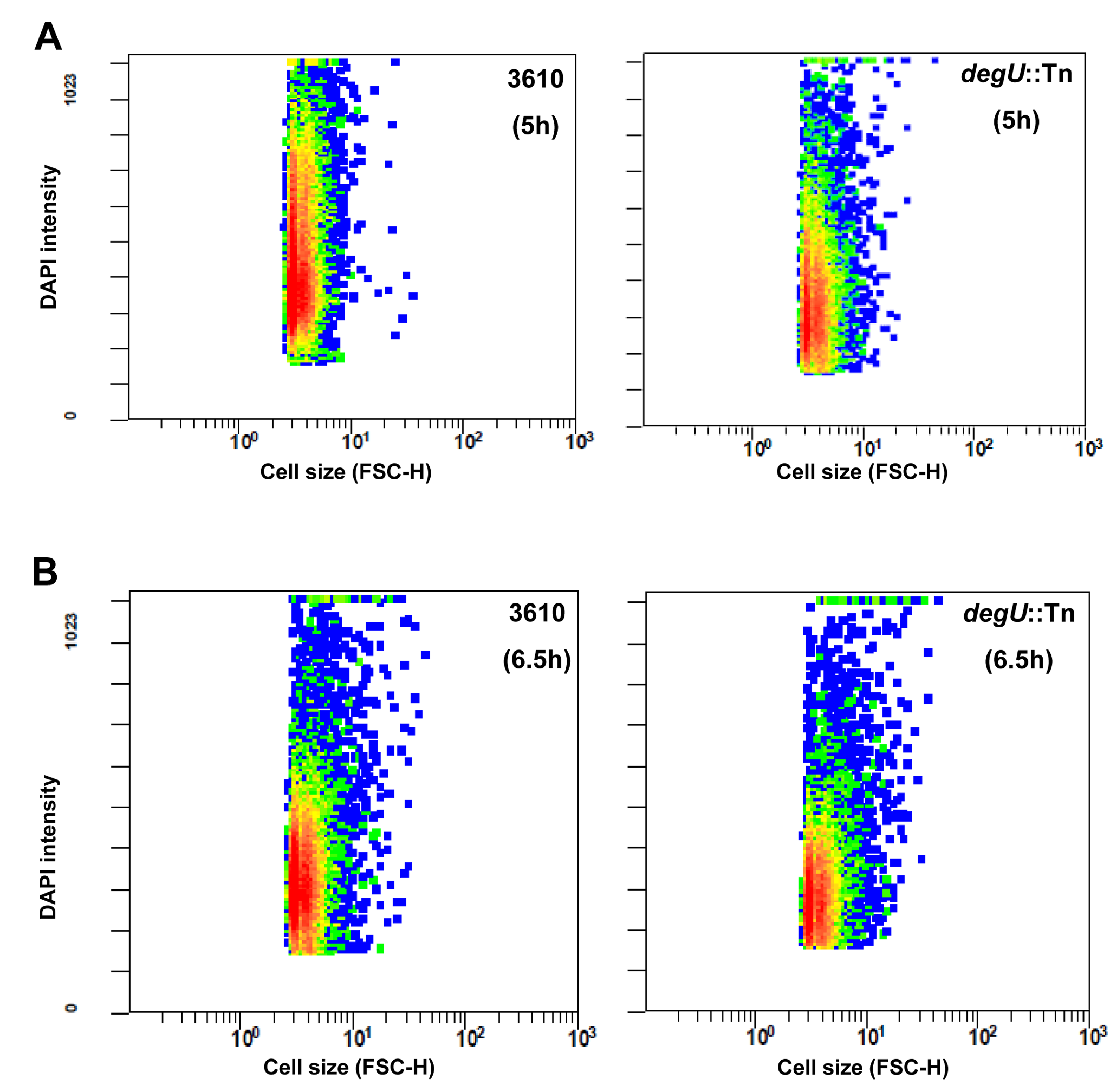

Supplement: Figure S7 — Flow cytometry analysis: density plots. It is shown the distribution of DAPI intensity versus cell size (FSC-H) into the population used in the analysis, from both wild type strain and degU::Tn mutant (GP229). Colours represent number of cells. A. 5 hours time point. B. 6.5 hours time point. (TIF) [file pone.0048716.s007.tif]

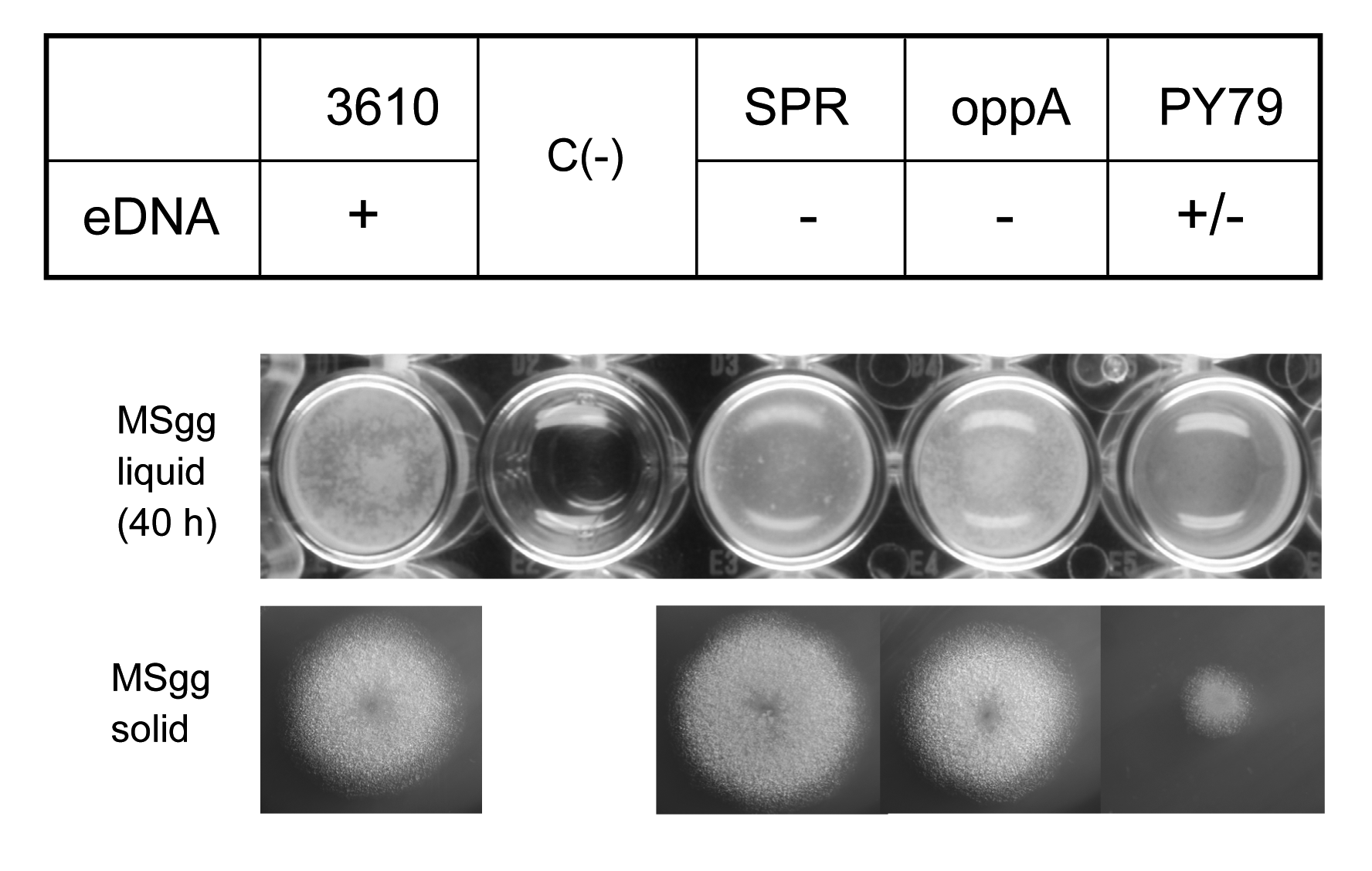

Supplement: Figure S8 — Effect of eDNA on biofilm formation. B. subtilis 3610, PY79, SPR-1 mutant (GP305) and oppA::mini-Tn10 (GP233) were grown in MSgg liquid medium at 30°C without shaking during 40 h, and in MSgg solid medium at 37°C 16 h. The presence of a biofilm is visualized as an opaque pellicle on the top of the liquid medium. Negative control refers to media without inoculation. (TIF) [file pone.0048716.s008.tif]

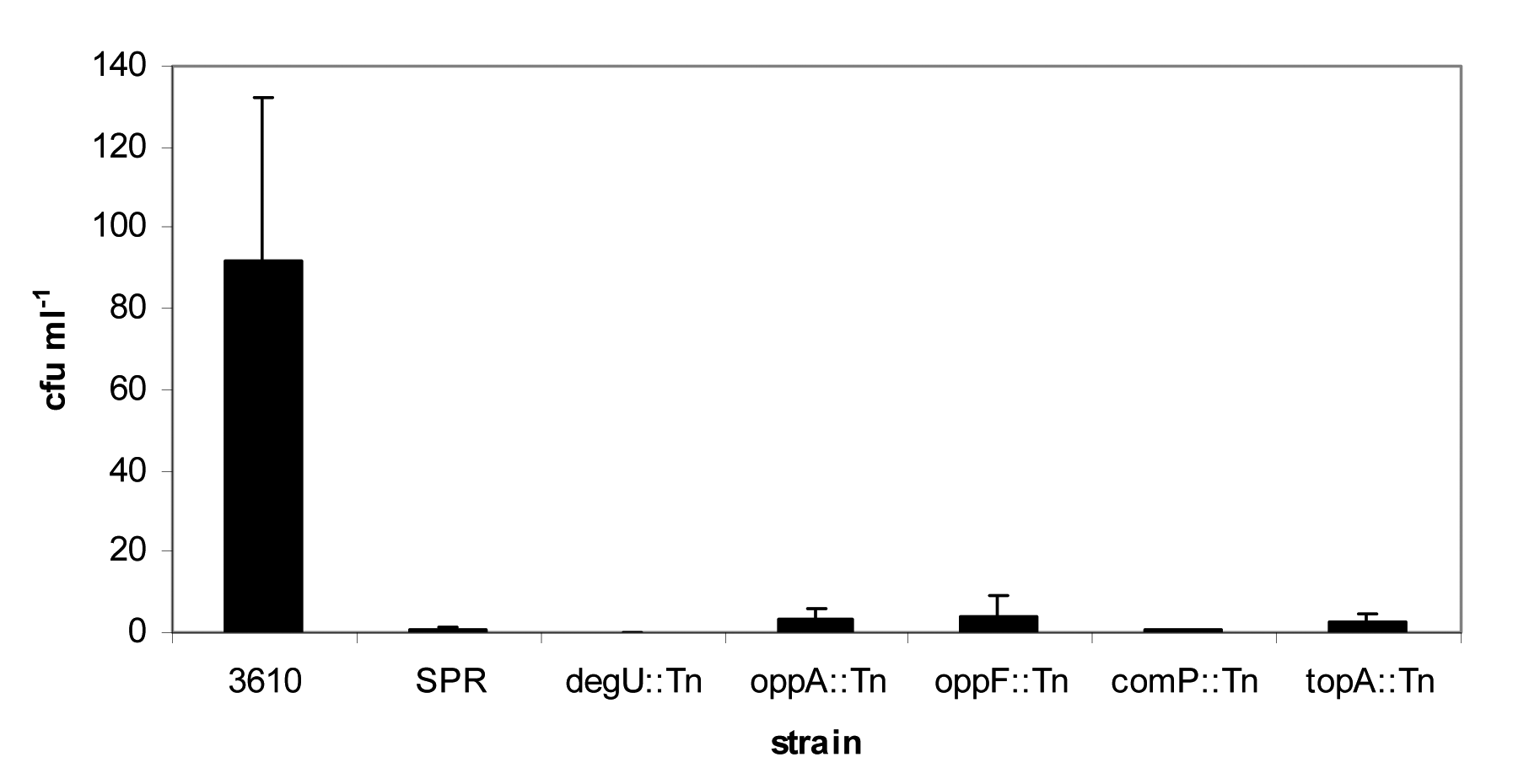

Supplement: Figure S9 — Competence assays in eDNA production mutants. 10 mg of genomic DNA with an antibiotic marker were transformed in several strains of B. subtilis and the colonies forming units were quantified to measure competence. (TIF) [file pone.0048716.s009.tif]
